# Blood Metabolite Signatures of Metabolic Syndrome in Two Cross-Cultural Older Adult Cohorts

**DOI:** 10.3390/ijms21041324

**Published:** 2020-02-16

**Authors:** Uma V. Mahajan, Vijay R. Varma, Chiung-Wei Huang, Yang An, Toshiko Tanaka, Luigi Ferrucci, Toru Takebayashi, Sei Harada, Miho Iida, Cristina Legido-Quigley, Madhav Thambisetty

**Affiliations:** 1 Clinical and Translational Neuroscience Section, Laboratory of Behavioral Neuroscience, National Institute on Aging, National Institutes of Health, Baltimore, MD 21224, USA; uvm2@case.edu (U.V.M.); vijay.varma01@gmail.com (V.R.V.); 2 Brain Aging and Behavior Section, Laboratory of Behavioral Neuroscience, National Institute on Aging, National Institutes of Health, Baltimore, MD 21224, USA; chiung-wei.huang@nih.gov (C.-W.H.); anya@grc.nia.nih.gov (Y.A.); 3 Translational Gerontology Branch, National Institute on Aging, NIH, Baltimore, MD 21224, USA; tanakato@mail.nih.gov (T.T.); ferruccilu@grc.nia.nih.gov (L.F.); 4 Department of Preventive Medicine and Public Health, Keio University School of Medicine, Tokyo 160-8582, Japan; ttakebayashi@a3.keio.jp (T.T.); seipeace2u@gmail.com (S.H.); mihoiida1029@gmail.com (M.I.); 5 Institute of Pharmaceutical Science, Kings College London, London SE19NH, UK; cristina.legido_quigley@kcl.ac.uk; 6 Steno Diabetes Center Copenhagen, 2820 Gentofte, Denmark

**Keywords:** metabolomics, metabolic syndrome, hypertension, diabetes, obesity, triglycerides, phosphatidylcholines, sphingomyelins, amino acids, ceramides, acylcarnitines

## Abstract

Metabolic syndrome (MetS) affects an increasing number of older adults worldwide. Cross-cultural comparisons can provide insight into how factors, including genetic, environmental, and lifestyle, may influence MetS prevalence. Metabolomics, which measures the biochemical products of cell processes, can be used to enhance a mechanistic understanding of how biological factors influence metabolic outcomes. In this study we examined associations between serum metabolite concentrations, representing a range of biochemical pathways and metabolic syndrome in two older adult cohorts: The Tsuruoka Metabolomics Cohort Study (TMCS) from Japan (*n* = 104) and the Baltimore Longitudinal Study of Aging (BLSA) from the United States (*n* = 146). We used logistic regression to model associations between MetS and metabolite concentrations. We found that metabolites from the phosphatidylcholines-acyl-alkyl, sphingomyelin, and hexose classes were significantly associated with MetS and risk factor outcomes in both cohorts. In BLSA, metabolites across all classes were uniquely associated with all outcomes. In TMCS, metabolites from the amino acid, biogenic amines, and free fatty acid classes were uniquely associated with MetS, and metabolites from the sphingomyelin class were uniquely associated with elevated triglycerides. The metabolites and metabolite classes we identified may be relevant for future studies exploring disease mechanisms and identifying novel precision therapy targets for individualized medicine.

## 1. Introduction

Over one third of adults in the United States suffer from metabolic syndrome, including conditions such as diabetes, obesity, hypertension, and hyperlipidemia [1]. The prevalence of these chronic diseases increases with age, and are associated with significant health care and other costs [2]. Evidence suggests that there are distinct ethnic and racial differences in prevalence, disease course, and risk factors associated with metabolic diseases [3]. Comparative population studies may enable better understanding of factors that underlie such differences. Insights from such comparative studies may also hold promise for the development of precision therapies for individualized medicine [4].

Japanese and American older adult cohorts have been extensively compared in relation to health outcomes because of known lifestyle and genetic differences. The Japanese population has also been studied as a comparison model of exceptional longevity [5]. Large, longitudinal studies including the Honolulu-Asia Aging Study (HAAS) [6] and the International Population Study on Macronutrients and Blood Pressure (INTERMAP) Study [7] have explored factors that affect metabolic risk as well as how cardiovascular risk factors may affect other health outcomes including dementia and cognitive impairment across Japanese, American, and other populations. Broadly, these studies have found a higher prevalence of metabolic diseases and conditions in the U.S compared to Japan, including higher body mass index (BMI) [8], diabetes [9], atherosclerosis [10], cardiovascular disease mortality [11], as well as higher blood cholesterol levels [12] and a differing prevalence of hypertension [13,14]. Lifestyle factors play key roles in mediating these differences [15]. While these cross-national studies provide insight into how environmental, cultural, and genetic factors may influence metabolic disease prevalence, they provide limited insight into the plausible mechanistic underpinnings of such population differences.

Metabolomics allows for the large-scale study of disparate small molecules that provide an indication of the biochemical products of cell processes. These tools can be used to better understand the functional and physiologic state of various tissues and can be used to measure alterations in biological pathways related to metabolic disease. The emergence of high-throughput metabolomics for analysis of molecular phenotypes, such as blood metabolite concentration levels, may enhance mechanistic understanding of how biological factors influence metabolic outcomes. Understanding the differences and similarities in the biological mechanisms underlying metabolic diseases across populations can provide important insights into treatment and prevention of these conditions. Recent studies have begun to explore cross-population and cross-ethnic differences in the metabolite signatures of various diseases. Two studies in Surinamese and Dutch cohorts [16] and South Asian and European cohorts [17], identified associations between type 2 diabetes and sphingolipids, acylcarnitines, and amino acids. Another study in overweight/obese Caucasians and African Americans identified differences in associations between small-molecule metabolites and metabolic hormones and overweight/obesity [18]. To our knowledge, no study has yet compared differences in serum metabolite concentrations in American and Japanese older adults.

In this study, we examined associations between serum metabolite concentrations, representing a diverse range of biochemical pathways and prevalent metabolic syndrome in two well-characterized older adult cohorts: The Tsuruoka Metabolomics Cohort Study (TMCS) from Japan and the Baltimore Longitudinal Study of Aging (BLSA) from the United States.

## 2. Results

### 2.1. Participants

The demographic characteristics of the BLSA participants and TMCS participants included in this study are summarized in Table 1. BLSA participants were significantly older and less likely to have never smoked. Measures used for assessing alcohol consumption and physical activity were different in BLSA and TMCS and therefore comparisons between groups are not presented. BLSA participants had higher levels of aspartate aminotransferase (AST) compared to TMCS participants while TMCS participants had higher levels of alanine aminotransferase (ALT) compared to BLSA participants. For both studies, the average AST and ALT were within the normal range [19]. BLSA and TMCS participants did not vary significantly in percent female, and in diet quality.

**Figure 1:**
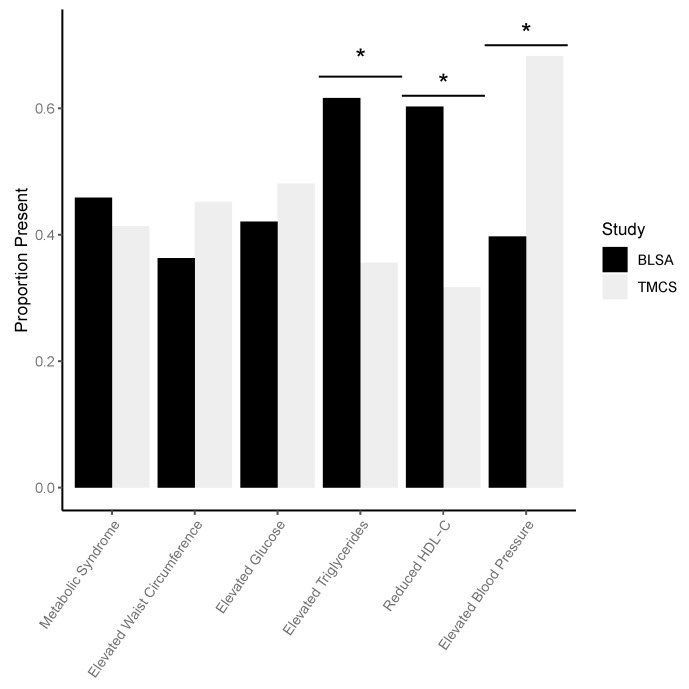
Distribution of metabolic syndrome (MetS) and individual MetS risk factors in each cohort. * indicates a significant difference at *p* < 0.05 between risk factor prevalence in BLSA compared to TMCS according to chi-square tests. BLSA: Baltimore Longitudinal Study of Aging; TMCS: Tsuruoka Metabolomics Cohort Study.

Figure 1 and Table 1 indicate differences by study in the proportion of participants with metabolic syndrome (MetS) and each individual MetS risk factor. BLSA participants and TMCS participants did not vary significantly in percentage with MetS, elevated waist circumference, and elevated fasting glucose. BLSA participants had a significantly higher percentage with elevated triglyceride level, a higher percentage with reduced high-density lipoproteins (HDL) cholesterol, and a lower percentage with elevated blood pressure. With regard to mean values of individual risk factors, BLSA participants had significantly larger waist circumference, lower HDL cholesterol, and lower systolic and diastolic blood pressure. With regard to drug use, BLSA participants had a higher percentage using lipid modifying therapies (LMTs) and a lower percentage using anti-hypertensive drugs; BLSA and TMCS participants did not vary in percent using diabetes medications.

### 2.2. Metabolite Concentrations: BLSA versus TMCS Differences

Supplementary Table S1 includes the mean metabolite concentrations for BLSA and TMCS and unadjusted and false discovery rate (FDR)-adjusted *p* values indicating whether differences between the two cohorts were significant. The majority of metabolites in each class were significantly different between the two cohorts. Out of 167 metabolites, 143 were significantly different between cohorts after FDR adjustment.

### 2.3. Associations with Metabolic Risk Factors

**Figure 2:**
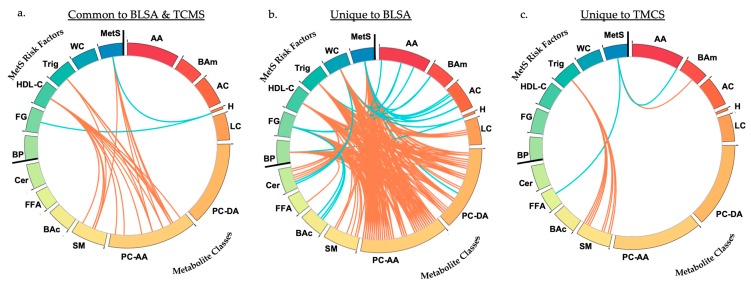
(**a**) Circos plot of overlapping significant associations in BLSA and TMCS; (**b**) Circos plot of significant associations unique to BLSA; (**c**) Circos plot of significant associations unique to TMCS. Outcomes, including MetS and individual risk factors, and metabolite classes are presented in bold and separated by black bold lines. Each color represents a different risk factor or metabolite class. BLSA: Baltimore Longitudinal Study of Aging; TMCS: Tsuruoka Metabolomics Cohort Study; AA: amino acids; BAm: biogenic amines; AC: acylcarnitines; H: hexoses; LC: lysophosphatidylcholines; PC-DA: phosphatidylcholines-diacyl; PC-AA: phosphatidylcholines-acyl-alkyl; SM: sphingomyelins; BAc: bile acids; FFA: free fatty acids; Cer: ceramides; BP: blood pressure; FG: fasting glucose; HDL-C: high-density lipoprotein cholesterol; Trig: triglycerides; WC: waist circumference; MetS: metabolic syndrome. Orange lines represent odds ratio < 1 at 0.05 FDR-adjusted significance level indicating that higher concentration of the metabolite is associated with a lower odds of the outcome. Turquoise lines represent odds ratio > 1 at 0.05 FDR-adjusted significance level indicating that higher concentration of the metabolite is associated with higher odds of the outcome.

In Table 2 and Table 3 and visualized in Figure 2a we present metabolites that were associated (FDR-adjusted *p* value) with either a diagnosis of MetS or with individual risk factors in both the BLSA and TMCS cohorts. Metabolites from the phosphatidylcholines-acyl-alkyl, sphingomyelin, and hexose classes were significantly associated with these outcomes in both cohorts. There were no metabolites significantly associated with waist circumference and blood pressure in both cohorts. In Supplementary Table S2 and visualized in Figure 2, we present metabolites that were associated (FDR adjusted *p* value) with either MetS or individual risk factors that are unique to BLSA (Supplementary Table S2a–f and Figure 2b) and TMCS (Supplementary Table S2g–h and Figure 2c), respectively. These were metabolites that were significantly associated with outcomes in one cohort and not the other. In BLSA, metabolites across all classes were uniquely associated with all outcomes (MetS and five individual risk factors). In TMCS, metabolites from the amino acid, biogenic amines, and free fatty acid classes were uniquely associated with MetS, and metabolites from the sphingomyelin class were uniquely associated with elevated triglycerides. There were no unique significant metabolite associations in TMCS for elevated glucose, elevated waist circumference, elevated blood pressure, and reduced HDL. All metabolite associations in both BLSA and TMCS are presented in Supplementary Files 3 (BLSA) and 4 (TMCS).

In Supplementary Files 5 (BLSA) and 6 (TMCS) we present all associations in both BLSA and TMCS from sensitivity analyses including additional covariates (lifestyle factors including smoking and diet quality) in multivariate logistic models. The majority of associations reported in Table 2 and Table 3 (20/22 associations) and in Supplementary Table S2 (174/200 associations) remained statistically significant in these sensitivity analyses; metabolites that remained significant after sensitivity analyses are indicated with a * in Table 2 and Table 3 and Supplementary Table S2. In Supplementary Files 7 (BLSA) and 8 (TMCS) we present sensitivity analyses indicating whether the association between metabolite concentrations and outcomes differed by sex. We include results (coefficient, standard error, *p* value, and FDR-adjusted *p* value) for the sex x metabolite term. In BLSA, the association between MetS/blood pressure and taurochenodeoxycholic acid (TCDCA) (FDR-corrected *p* value = 0.01909 and 0.02918, respectively) was significantly different by sex, and the association between fasting glucose and cis-11-Eicosenoic acid (c-C20:1w9) (FDR- corrected *p* value = 0.03907) was also significantly different by sex. In TMCS, the association between blood pressure and the acylcarnitines: tetradecenoylcarnitine (C14:1), tetradecadienoylcarnitine (C14:2), propionylcarnitine (C3), and butyrylcarnitine (C4) (FDR-corrected *p* value = 0.0495 for all four metabolites) were significantly different by sex.

## 3. Discussion

Participants in the American and Japanese cohorts examined in this study did not vary in percentage of individuals with MetS, however, the cohorts varied significantly across individual vascular risk factors. The American cohort had a significantly higher percentage of individuals with elevated triglycerides and reduced HDL despite a significantly higher percentage of individuals being on lipid modifying treatments (LMTs). The Japanese cohort had significantly elevated blood pressure despite a significantly higher percentage of individuals being on anti-hypertensive medications. There were no differences in percentage of individuals with high waist circumference or percent of individuals with elevated fasting glucose.

Prior studies have reported significant lifestyle differences between American and Japanese cohorts, including a healthier diet—lower fat consumption and higher omega-3 fatty acids—as well as increased physical activity across the lifespan among Japanese [7,20]. Diet is an important component of lifestyle and a potential mediator of cohort differences. In this study, we assessed diet using the Dietary Approaches to Stop Hypertension (DASH) score- an indicator of a dietary pattern rich in fruits, vegetables, and low-fat dairy produce and low in meats and sweets. This dietary pattern has been shown to protect against risk factors of metabolic syndrome [21] in multiple ethnic groups [22,23]. We did not see significant differences in DASH scores between the BLSA and TMCS cohorts, suggesting that diet may not be a significant driver of differences in vascular risk profiles and may explain the lack of a difference in MetS between the American and Japanese cohorts included in this study. We additionally found that smoking rates were higher in the American cohort despite prior studies indicating that Japanese men have higher smoking rates than American men [24].

Despite similarities in the prevalence of MetS in the BLSA and TMCS cohorts as well as similarities in diet, we found differences between cohorts for over 85% of metabolites included in the analyses. These differences are likely a reflection of both biological and environmental variability between the cohorts.

We identified significant cohort differences across concentrations of the majority of metabolites as well as differences in lifestyle factors, including smoking prevalence. We additionally identified significant differences in important biologic factors, including AST and ALT, that have been shown to be elevated in nonalcoholic fatty liver disease (NAFLD), a disorder with a bi-directional association with MetS and individual risk factors [25,26]. Despite these differences, it is striking that there is a distinct subset of metabolites that are associated with MetS and individual risk factors in both cohorts. These metabolites included phosphatidylcholines-acyl-alkyls, sphingomyelins, and hexoses, suggesting that metabolites in these three classes may represent fundamental markers of MetS and vascular risk. Serum concentrations of these metabolites may be important indicators of disease mechanisms that may also provide insights into effective treatments.

A large body of work has shown that phosphatidylcholines are important regulators of energy metabolism [27], and perturbations in phosphatidylcholine levels may be associated with mitochondrial dysfunction and a number of metabolic diseases including diabetes and cardiovascular disease [27,28,29], as well as NAFLD [30]. Phosphatidylcholines-acyl-alkyls have specifically been shown to be associated with obesity and metabolic syndrome in a Canadian cohort [31]. Two phosphatidylcholine metabolites identified in this study (PCaec34:3 and PCaec36:3) were also associated with diabetes mellitus progression and risk of onset of diabetes in Taiwanese [32] and Korean [33] cohorts, respectively. Interestingly, we previously reported that two metabolites also identified in this study (PC ae C34:2 and PC ae C36:3) were among a panel of metabolites that discriminated between Alzheimer’s disease and control samples [34]. These converging results suggest that aberrations in specific phosphatidylcholines-acyl-alkyls may be intrinsic to diseases associated with vascular risk and, therefore, potentially important targets for disease intervention [35].

Our results additionally indicate that sphingomyelins are associated with MetS (i.e., SM C16:0) as well as with individual vascular risk factors (i.e., SM C16:0, SM C16:1, SM C24:1) in both cohorts. Similar to phosphatidylcholines-acyl-alkyls, sphingomyelins are major components of cell membranes and participate in cell-signaling pathways. Elevated levels of sphingomyelins have been shown to play a critical role in cardiovascular dysfunction including insulin resistance, atherosclerosis, and cardiomyopathy [36]. While levels of SM C16:0 were not shown to be associated with the clinical features of MetS in a young Japanese sample [37], SM C16:0 was shown to be associated with insulin resistance in a Southern European cohort at high risk for cardiovascular disease [38]. Sphingomyelins likely play a significant role in chronic inflammation and may therefore be implicated in multiple metabolic diseases, including those of old age such as dementia [34,39,40,41].

Our finding that hexose (the sum of all hexoses, which is predominantly glucose) was associated with MetS and fasting glucose was to be expected considering that fasting glucose and the serum concentration of total hexoses are highly correlated values. Additionally, increased concentration of hexoses was uniquely associated with an increased odds of elevated waist circumference and elevated blood pressure in BLSA only. This suggests that in the BLSA cohort, elevated peripheral hexoses, likely representing predominantly glucose, may be a more significant indicator of vascular risk than in the TMCS cohort. Prior work has suggested that increased serum hexoses may indicate poor glycemic control and insulin resistance [42].

We additionally found metabolites significantly associated with outcomes that were cohort specific.

One amino acid, valine, was associated with increased odds of MetS in TMCS. A number of recent studies have indicated the emerging importance of essential and non-essential amino acids in the development of obesity and diabetes mellitus [43,44,45,46,47]. Valine in particular was one of three essential amino acids associated with obesity and visceral obesity in a Japanese volunteer sample (age range 20–60) [48]; valine was also identified as associated with MetS in another Japanese sample (average age: 55) [43]. Interestingly, a previous study using plasma samples from post-menopausal women in the TMCS assayed metabolites using capillary electrophoresis time-of-flight mass spectrometry (CE-TOF MS) and reported higher concentrations of the branched chain amino acids (BCAAs) including valine in participants with MetS. [49]. Our observation of a similar association between serum concentration of valine and MetS in the TMCS cohort using LC-MS/MS confirms the prior findings reported by Iida et al. [49]. Together with prior studies showing that valine is associated with vascular risk factors [50] and NAFLD [30] even in non-Japanese populations, our work suggests the importance of exploring the role of valine as a biomarker of vascular risk as well as a focus of targeted therapies.

A biogenic amine, alpha-aminoadipic acid (alpha-AAA), was associated with increased odds of MetS and increased triglycerides in the BLSA cohort. A study utilizing LC-MS/MS in the Framingham Heart Study found that this metabolite was positively associated with risk of developing diabetes, and individuals with alpha-AAA concentrations in the top quartile had greater than a four-fold risk of developing diabetes [51].

There are several important limitations to our work. In this study, the Japanese group had a more restricted age range and were all recruited from the same city and prefecture in Japan whereas the United States (U.S.) group was more diverse, which may have partially driven differences in total number of unique associations between metabolites and outcomes. While the Japanese group is a more population-representative sample, the BLSA is a predominantly Caucasian sample of highly educated and relatively healthy older adults. Additionally, our metabolomic assays only detected a limited number of classes of metabolites in the serum metabolome. Also, we were only able to include lifestyle factors derived from comparable questionnaires between studies. Important lifestyle variables including physical activity and alcohol consumption were not measured similarly in the BLSA and TMCS studies and therefore could not be compared or included in our analyses. Finally, the cross-sectional nature of our analyses does not allow us to assess how alterations in metabolite concentrations over time may be related to the risk of MetS.

## 4. Methods and Materials

### 4.1. Participants

The National Institute on Aging’s (NIA) BLSA is one of the longest running scientific studies of human aging in the U.S. [52]. The individuals in this study were participants in the neuroimaging substudy of the BLSA [53]. Written informed consent was obtained at each visit for all BLSA participants. The BLSA study protocol has ongoing approval from the Institutional Review Board of the National Institute of Environmental Health Science, National Institutes of Health (“Early Markers of Alzheimer’s Disease (BLSA)”, Institutional Review Board number 2009-074). The Tsuruoka Metabolomics Cohort Study (TMCS) is a population-based study of residents from Tsuruoka City, Yamagata Prefecture, Japan that began in 2012. The individuals in this study were a subset from the baseline survey participants of the TMCS [54]. Written informed consent was obtained for all TMCS participants. The TMCS study protocol has ongoing approval from the Medical Ethics Committee of the School of Medicine, Keio University, Tokyo, Japan (Approval No. 20110264; original date of approval: 12/06/2011; latest update: 12/02/2019).

### 4.2. Blood Samples

Blood serum samples were collected from BLSA participants at the NIA Clinical Research Unit in Harbor Hospital, Baltimore. Details on serum collection and processing have been published previously [55]. The sample included 146 participants.

Blood serum samples were collected from TMCS participants during annual health check-ups. Details on serum collection and processing have been published previously [49,54]. The sample included 104 participants.

Additional details on sample collection are included in Supplementary Text S1.

### 4.3. Other Outcomes

For the BLSA and TMCS samples, a description of data collection details for plasma/serum triglycerides, glucose, HDL-C, waist circumference, and systolic and diastolic blood pressures is included in Supplementary Text S1.

### 4.4. Metabolites

After data cleaning (see Statistical Analyses section for details), a total of 167 metabolites across 11 categories were assayed for BLSA and TMCS serum samples. Metabolite categories included amino acids (21 metabolites), biogenic amines (10 metabolites), acylcarnitines (12 metabolites), hexoses (1 metabolite; includes the sum of all hexoses and is 90–95% glucose), lysophosphatidylcholines (11 metabolites), phosphatidylcholines with diacyl residue (34 metabolites), phosphatidylcholines with acyl-alkyl residue (36 metabolites), sphingomyelins (14 metabolites), bile acids (10 metabolites), free fatty acids (8 metabolites), and ceramides (10 metabolites).

Quantitative metabolomics in BLSA serum samples was performed on the Biocrates AbsoluteIDQ p180 platform. Details on the assays have been published previously [34]. The quantification of bile acids and targeted lipid metabolomics in the BLSA sample are described in detail in Supplementary Text S1.

Quantitative metabolomics in TMCS serum samples was also performed with the Biocrates AbsoluteIDQ p180 platform. For additional metabolites including bile acids and additional lipids, assays were performed with the Biocrates MxP^®^ Quant 500 kit described in detail in Supplementary Text S1.

P180 and Q500 platforms included redundant metabolites across multiple metabolite categories including amino acids, acylcarnitines, sphingomyelins, phosphatidylcholines, hexoses, and biogenic amines. The correlation (Pearson’s r) between the two values (i.e., P180 and Q500 estimated metabolite concentrations on the same participant blood sample) varied between 0.465–0.991 (*p* values all < 0.001) with over 75% of metabolites with a correlation of > 0.900. For redundant metabolites we preferentially used P180 data, similar to the procedure used in BLSA.

Concentration of each metabolite is indicated in μM.

### 4.5. Metabolic Syndrome Risk Factors

Metabolic syndrome (MetS) was defined using the Third Adults Treatment Panel of the National Cholesterol Education Program (NCEP ATP III) criteria, revised by the American Heart Association and National Heart, Lung, and Blood Institute (AHA/NHLBI). Briefly, a diagnosis of MetS was made in the presence of at least three of five criteria/risk factors, including elevated waist circumference, elevated fasting glucose, elevated triglyceride levels, reduced HDL cholesterol, and elevated blood pressure.

Elevated waist circumference was defined in BLSA as greater than 40 inches in males, and greater than 35 inches in females. For the TMCS participants, the criterion was modified for a Japanese population and was defined as greater than or equal to 90 cm (35.433 inches) in males, and greater than or equal to 80 cm (31.496 inches) in females [49].

Elevated fasting glucose was defined as greater than or equal to 100 mg/dl, or the use of prescription diabetes medications.

Elevated triglyceride level was defined as greater than or equal to 150 mg/dl, or the use of prescription lipid modifying treatments (LMTs).

Reduced HDL cholesterol was defined as less than to 40 mg/dl in males, less than 50 mg/dl in females, or the use of prescription LMTs medications.

Elevated blood pressure was defined as greater than or equal to 130 mm Hg systolic blood pressure, greater than or equal to 85 mm Hg diastolic blood pressure, or the use of prescription anti-hypertensive medications.

Recording of prescription medications in both cohorts was based on self-reported information collected through a standardized questionnaire.

### 4.6. Additional Demographic Variables and Covariates

Smoking status and diet quality were used in the sensitivity analyses described below. Smoking history was dichotomized as ‘never smoker’ vs. ‘current/former smoker.’ Diet quality was measured using the Dietary Approaches to Stop Hypertension (DASH) score [56]. The DASH score indicates adherence to the DASH dietary pattern by measuring consumption of nine target nutrients including total fat, saturated fat, protein, fiber, cholesterol, calcium, potassium, and magnesium. We excluded magnesium as this target was not included in TMCS data collection. The DASH score therefore was the sum of eight nutrient components with higher values indicating a higher quality diet.

Liver function tests, physical activity, and alcohol consumption were included as additional demographic variables to describe BLSA and TMCS participants. Liver function tests included aspartate aminotransferase (AST) and alanine aminotransferase (ALT), both shown to be elevated in non-alcohol fatty liver disease [57] and associated with MetS, insulin resistance, hypertension, and dyslipidemia [58,59]. Physical activity (PA) was measured using two different questionnaires in BLSA and TMCS. The PA questionnaire in BLSA has been described previously [60]; total PA was determined by summing physical activity across three intensity categories (low, medium, high) and multiplying the hours spent in each activity/week by the assigned metabolic equivalent (MET) value. The PA questionnaire used in TMCS has been described previously [61]; total PA was determined by summing all domains of activity (occupational activity, leisure time activity, sleeping, and other activities) and multiplying the hours spent in each activity/week by the assigned MET value. PA in both studies was summarized as ‘METs/week.’ Alcohol consumption in BLSA was dichotomized as ‘never’ vs. ‘ever drank’ any alcoholic beverages in the past 12 months. Alcohol consumption in TMCS was dichotomized as ‘current drinker’ vs. ‘never/ex-drinker.’

### 4.7. Statistical Analyses

We first excluded all metabolites with > 30% missing values (i.e., values indicated as lower than the limit of detection (LOD)) resulting in 167 total metabolites that were included in our analyses. We then imputed all missing values as the LOD threshold/2. Finally, we excluded all outliers defined as values outside the 3 interquartile region (IQR) (i.e., 3 × IQR).

Next, we compared demographic characteristics as well as prevalence of MetS and its individual risk factors (as well as mean values) between BLSA and TMCS using two-sample t-tests for continuous variables and chi-squared tests for categorical variables. We compared differences in metabolite concentrations between BLSA and TMCS. To account for multiple comparisons, we corrected for each of the 11 metabolite classes separately using false discovery rate (FDR)-adjusted *p* value [62]. We then used multivariate logistic regression models to explore the associations between the binary indicators of MetS (i.e., present/absent) and its five individual risk factors with metabolite concentrations. These models tested whether individuals with MetS or the five individual risk factors had different metabolite concentrations compared to control participants (i.e., without MetS or the five individual risk factors). This included six separate models, each with the following outcomes/dependent variables: MetS, elevated waist circumference, elevated fasting glucose, elevated triglycerides, reduced HDL cholesterol, and elevated blood pressure. Metabolite concentrations, which were the predictors/independent variables, were IQR normalized by subtracting the median from the metabolite value and dividing by the IQR. Model results indicate an increase or decrease in the odds of having MetS or an individual risk factor associated with a one-unit increase in the normalized metabolite concentration(s). Sex and age at blood draw were included as covariates in the model. We performed two sensitivity analyses: First, we explored associations controlling for additional lifestyle factors including smoking status and diet quality. We ran multivariate logistic models including both covariates, in addition to age and sex. Second, we explored associations in the main model including an additional sex interaction term (sex x metabolite) to explore whether the association between the metabolite and outcome differed by sex. For all multivariate models, we reported odds ratios with 95% confidence intervals. Type I error level was set to 0.05 for unadjusted *p* values. We also accounted for multiple comparisons using FDR-adjusted *p* values as described above. We used SAS version 9.4 (Cary, NC, United States) for all data analyses and R Studio 1.1.453 for data visualization.

## 5. Conclusions

Our results indicate the importance of identifying similarities as well as differences in associations between alterations in the serum metabolome and metabolic syndrome across ethnic groups. We identified several classes of metabolites that may be intrinsic to MetS, independent of significant differences across two ethnically distinct cohorts. Biologic pathways associated with these metabolites (e.g., phosphatidylcholines-acyl-alkyl, sphingomyelin, and hexoses) may be targets for future studies exploring unique disease mechanisms as well as for identifying novel targets for person-centered pharmacotherapeutic interventions. 

## Figures and Tables

**Table 1:** Participant demographics.

Demographic Variable	BLSA (*n* = 146)	TMCS (*n* = 104)	*p* Value
Female, *n* (%)	77 (52.74)	66 (63.46)	0.0913
White, *n* (%)	112 (76.71)	-	-
Storage time (years), Mean (SD)	4.36 (2.33)	-	-
Age (years), Mean (SD)	76.08 (8.54)	69.67 (2.57)	<0.0001
Never smoke, *n* (%)	74 (50.68)	74 (71.15)	0.0012
DASH (no Mg), Mean (SD)	2.62 (0.78)	2.80 (1.21)	0.1459
Drinker, *n* (%)	128 (88.28)	41 (39.42)	*
Physical Activity (METs/week), Mean (SD)	77.77 (66.01)	17.77 (15.32)	*
**Metabolic syndrome**			
Metabolic syndrome, *n* (%)	67 (45.89)	43 (41.35)	0.4756
**Waist circumference**			
Elevated waist circumference, *n* (%)	53 (36.3)	47 (45.19)	0.1573
Waist circumference (in), Mean (SD)	35.82 (4.65)	32.43 (3.37)	<0.0001
**Fasting glucose**			
Elevated fasting glucose, *n* (%)	61 (42.07)	50 (48.08)	0.3469
Fasting glucose (mg/dL), Mean (SD)	101.04 (20.68)	102.3 (15.03)	0.5794
Diabetes drug use, *n* (%)	14 (9.72)	9 (8.65)	0.7747
**Triglycerides**			
Elevated triglyceride level, *n* (%)	90 (61.64)	37 (35.58)	<0.0001
Triglyceride level (mg/dL), Mean (SD)	97.88 (42.15)	102.4 (60.05)	0.5092
LMT use, *n* (%)	82 (56.94)	29 (27.88)	<0.0001
**HDL cholesterol**			
Reduced HDL cholesterol, *n* (%)	88 (60.27)	33 (31.73)	<0.0001
HDL cholesterol (mg/dL), Mean (SD)	61.36 (15.12)	68.97 (23.76)	0.0046
LMT drug use, *n* (%)	82 (56.94)	29 (27.88)	<.0001
**Blood Pressure**			
Elevated Blood Pressure, *n* (%)	58 (39.73)	71 (68.27)	<0.0001
SBP (mm Hg), Mean (SD)	114.32 (12.88)	130.87 (16.95)	<0.0001
DBP (mm Hg), Mean (SD)	64.32 (9.06)	74.83 (10.28)	<0.0001
Hypertension drug use, *n* (%)	44 (30.56)	46 (44.23)	0.0271
**Liver Enzymes**			
Aspartate aminotransferase (AST) (U/L), Mean (SD)	27.11 (10.25)	20.57 (11.17)	0.0059
Alanine aminotransferase (ALT) (U/L), Mean (SD)	21.73 (8.88)	24.59 (7.37)	<0.0001

* The questionnaires used to measure alcohol consumption and physical activity were different in BLSA compared to TMCS therefore comparisons between groups are not presented. Two-sample t-tests were used to compare continuous variables and chi-squared tests were used to compare categorical variables between the two cohorts. BLSA: Baltimore Longitudinal Study of Aging; TMCS: Tsuruoka Metabolomics Cohort Study; SD: standard deviation; LMT: lipid modifying treatment; METs: metabolic equivalents; DASH: Dietary Approaches to Stop Hypertension score; SBP: systolic blood pressure; DBP: diastolic blood pressure; HDL: high-density lipoproteins; mg/dL: milligrams/deciliter; in: inches; mm HG: millimeters mercury; U/L: units/liter.

**Table 2:** Adjusted significant associations in both BLSA and TMCS: MetS.

Metabolite	Category	BLSA					TMCS					Direction of Assoc.
		Odds Ratio Estimate	Lower 95% CI	Upper 95% CI	*p* Value	FDR-Adjusted *p* Value	Odds Ratio Estimate	Lower 95% CI	Upper 95% CI	*p* Value	FDR-Adjusted *p* Value	
Met S												
H1 *	Hexoses	3.4	1.863	6.207	<0.0001	<0.0001	1.918	1.121	3.28	0.0174	0.01743	+
PC ae C34:2 *	Phosphatidylcholines-acyl-alkyl	0.246	0.129	0.468	<0.0001	0.00014	0.24	0.108	0.533	<0.0001	0.01429	-
PC ae C34:3 *	Phosphatidylcholines-acyl-alkyl	0.249	0.128	0.484	<0.0001	0.00019	0.285	0.136	0.598	<0.0001	0.01429	-
PC ae C36:3 *	Phosphatidylcholines-acyl-alkyl	0.221	0.112	0.436	<0.0001	0.00012	0.326	0.163	0.654	0.0016	0.01429	-
PC ae C38:2 *	Phosphatidylcholines-acyl-alkyl	0.218	0.114	0.417	<0.0001	<0.0001	0.298	0.142	0.623	0.0013	0.01429	-
SM C16:0 *	Sphingomyelins	0.339	0.191	0.602	0.0002	0.00299	0.369	0.196	0.697	0.0021	0.02956	-

* Associations that remain significant after adjustment with additional covariates smoking and DASH score. Multivariate logistic regression models were used to test for significance. Negative (-) Direction of Association (Assoc.): odds ratio < 1 at 0.05 significance level indicating that higher concentration of the metabolite is associated with lower odds of the outcome/risk factor. Positive (+) Direction of Association: odds ratio > 1 at 0.05 significance level indicating that higher concentration of the metabolite is associated with higher odds of the outcome/risk factor. BLSA: Baltimore Longitudinal Study of Aging; TMCS: Tsuruoka Metabolomics Cohort Study; FDR: false discovery rate; CI: confidence interval.

**Table 3:** Adjusted significant associations in both BLSA and TMCS: glucose, HDL, and triglyceride levels.

Metabolite	Category	BLSA					TMCS					Direction of Assoc.
		Odds Ratio Estimate	Lower 95% CI	Upper 95% CI	*p* Value	FDR-Adjusted *p* Value	Odds Ratio Estimate	Lower 95% CI	Upper 95% CI	*p* Value	FDR-Adjusted *p* Value	
Glucose												
H1*	Hexoses	24.297	7.99	73.892	<0.0001	<0.0001	70.821	12.211	410.741	<0.0001	<0.0001	+
HDL												
PC ae C32:1 *	Phosphatidylcholines-acyl-alkyl	0.271	0.142	0.515	<0.0001	0.00125	0.405	0.208	0.788	0.0078	0.04004	-
PC ae C34:2 *	Phosphatidylcholines-acyl-alkyl	0.417	0.241	0.721	0.0018	0.01105	0.286	0.129	0.637	0.0022	0.03163	-
PC ae C34:3 *	Phosphatidylcholines-acyl-alkyl	0.455	0.263	0.785	0.0047	0.01369	0.253	0.109	0.586	0.0013	0.03163	-
PC ae C36:3 *	Phosphatidylcholines-acyl-alkyl	0.363	0.2	0.657	0.0008	0.00609	0.331	0.157	0.695	0.0035	0.03163	-
PC ae C38:2 *	Phosphatidylcholines-acyl-alkyl	0.285	0.156	0.52	<0.0001	0.00125	0.309	0.143	0.67	0.0029	0.03163	-
PC ae C40:1 *	Phosphatidylcholines-acyl-alkyl	0.474	0.283	0.794	0.0046	0.01349	0.388	0.197	0.765	0.0063	0.03755	-
PC ae C42:3 *	Phosphatidylcholines-acyl-alkyl	0.433	0.243	0.773	0.0046	0.01349	0.385	0.195	0.761	0.006	0.03755	-
PC ae C44:4 *	Phosphatidylcholines-acyl-alkyl	0.561	0.357	0.88	0.0119	0.02359	0.402	0.2	0.807	0.0104	0.04673	-
SM C16:0 *	Sphingomyelins	0.407	0.236	0.703	0.0013	0.01741	0.293	0.144	0.594	<0.0001	0.00934	-
SM C16:1 *	Sphingomyelins	0.448	0.245	0.816	0.0087	0.02928	0.419	0.218	0.805	0.0091	0.04231	-
SM C24:1*	Sphingomyelins	0.42	0.237	0.745	0.003	0.02039	0.376	0.194	0.73	0.0039	0.02695	-
Triglycerides												
PC ae C34:2 *	Phosphatidylcholines-acyl-alkyl	0.437	0.252	0.758	0.0032	0.01378	0.3	0.141	0.641	0.0019	0.03356	-
PC ae C34:3	Phosphatidylcholines-acyl-alkyl	0.535	0.313	0.914	0.0221	0.04221	0.294	0.138	0.63	0.0016	0.03356	-
PC ae C38:2 *	Phosphatidylcholines-acyl-alkyl	0.301	0.165	0.55	<0.0001	0.00343	0.344	0.168	0.708	0.0037	0.04498	-
SM C16:0	Sphingomyelins	0.446	0.26	0.766	0.0034	0.04703	0.328	0.168	0.638	0.001	0.00722	-

* Associations that remain significant after adjustment with additional covariates smoking and DASH score. Multivariate logistic regression models were used to test for significance. Negative (-) Direction of Association: odds ratio < 1 at 0.05 significance level indicating that higher concentration of the metabolite is associated with lower odds of the outcome/risk factor. Positive (+) Direction of Association: odds ratio > 1 at 0.05 significance level indicating that higher concentration of the metabolite is associated with higher odds of the outcome/risk factor. BLSA: Baltimore Longitudinal Study of Aging; TMCS: Tsuruoka Metabolomics Cohort Study; FDR: false discovery rate; CI: confidence interval.

## Supplementary Materials

Supplementary materials can be found at https://www.mdpi.com/1422-0067/21/4/1324/s1.

## References

[1] Aguilar M., Bhuket T., Torres S., Liu B., Wong R.J. (2015). Prevalence of the metabolic syndrome in the United States, 2003–2012. JAMA.

[2] Birnbaum H.G., Mattson M.E., Kashima S., Williamson T.E. (2011). Prevalence rates and costs of metabolic syndrome and associated risk factors using employees’ integrated laboratory data and health care claims. J. Occup. Environ. Med..

[3] Moore J.X., Chaudhary N., Akinyemiju T. (2017). Metabolic Syndrome Prevalence by Race/Ethnicity and Sex in the United States, National Health and Nutrition Examination Survey, 1988–2012. Prev. Chronic Dis..

[4] Clish C.B. (2015). Metabolomics: An emerging but powerful tool for precision medicine. Cold Spring Harb. Mol. Case Stud..

[5] Pignolo R.J. (2019). Exceptional Human Longevity. Mayo Clin. Proc..

[6] Kalmijn S., Foley D., White L., Burchfiel C.M., Curb J.D., Petrovitch H., Ross G.W., Havlik R.J., Launer L.J. (2000). Metabolic cardiovascular syndrome and risk of dementia in Japanese-American elderly men. The Honolulu-Asia aging study. Arterioscler. Thromb. Vasc. Biol..

[7] Ueshima H., Okayama A., Saitoh S., Nakagawa H., Rodriguez B., Sakata K., Okuda N., Choudhury S.R., Curb J.D., Group I.R. (2003). Differences in cardiovascular disease risk factors between Japanese in Japan and Japanese-Americans in Hawaii: The INTERLIPID study. J. Hum. Hypertension.

[8] Yeom J., Kim J.K., Crimmins E.M. (2009). Factors Associated with Body Mass Index (BMI) Among Older Adults: A Comparison Study of the U.S., Japan, and Korea. Hanguk Nonyonhak.

[9] Obika M., Trence D.L. (2010). Comparison of type 2 diabetes care in the United States and Japan. Endocr. Pract..

[10] Fujiyoshi A., Miura K., Ohkubo T., Kadowaki T., Kadowaki S., Zaid M., Hisamatsu T., Sekikawa A., Budoff M.J., Liu K. (2014). Cross-sectional comparison of coronary artery calcium scores between Caucasian men in the United States and Japanese men in Japan: The multi-ethnic study of atherosclerosis and the Shiga epidemiological study of subclinical atherosclerosis. Am. J. Epidemiol..

[11] Comstock G.W., Suzuki T., Stone R.W., Crumrine J.L., Johnson D.H., Sakai Y., Matsuya T., Sasaki S. (1985). Cardiovascular risk factors in American and Japanese executives. Telecom Health Research Group. J. R. Soc. Med..

[12] Namekata T., Moore D., Knopp R., Marcovina S., Perrin E., Hughes D., Suzuki K., Mori M., Sempos C., Hatano S. (1996). Cholesterol levels among Japanese Americans and other populations: Seattle Nikkei Health Study. J. Atheroscler. Thromb..

[13] Imazu M., Sumida K., Yamabe T., Yamamoto H., Ueda H., Hattori Y., Miyauchi A., Hara H., Yamakido M. (1996). A comparison of the prevalence and risk factors of high blood pressure among Japanese living in Japan, Hawaii, and Los Angeles. Public Health Rep..

[14] Saito Y., Davarian S., Takahashi A., Schneider E., Crimmins E.M. (2015). Diagnosis and Control of Hypertension in the Elderly Populations of Japan and the United States. Int. J. Popul. Stud..

[15] Shiwa M., Yoneda M., Nakanishi S., Oki K., Yamane K., Kohno N. (2015). Japanese lifestyle during childhood prevents the future development of obesity among Japanese-Americans. PLoS ONE.

[16] van Valkengoed I.G.M., Argmann C., Ghauharali-van der Vlugt K., Aerts J., Brewster L.M., Peters R.J.G., Vaz F.M., Houtkooper R.H. (2017). Ethnic differences in metabolite signatures and type 2 diabetes: A nested case-control analysis among people of South Asian, African and European origin. Nutr. Diabetes.

[17] Tillin T., Hughes A.D., Wang Q., Wurtz P., Ala-Korpela M., Sattar N., Forouhi N.G., Godsland I.F., Eastwood S.V., McKeigue P.M. (2015). Diabetes risk and amino acid profiles: Cross-sectional and prospective analyses of ethnicity, amino acids and diabetes in a South Asian and European cohort from the SABRE (Southall And Brent REvisited) Study. Diabetologia.

[18] Patel M.J., Batch B.C., Svetkey L.P., Bain J.R., Turer C.B., Haynes C., Muehlbauer M.J., Stevens R.D., Newgard C.B., Shah S.H. (2013). Race and sex differences in small-molecule metabolites and metabolic hormones in overweight and obese adults. OMICS.

[19] Liver Function Tests. https://www.mayoclinic.org/tests-procedures/liver-function-tests/about/pac-20394595.

[20] Senauer B., Gemma M., Minnesota U.O. (2006). Why Is the Obesity Rate So Low in Japan and High in the U.S.? Some Possible Economic Explanations.

[21] Akhlaghi M. (2019). Dietary Approaches to Stop Hypertension (DASH): Potential mechanisms of action against risk factors of the metabolic syndrome. Nutr. Res. Rev..

[22] Joyce B.T., Wu D., Hou L., Dai Q., Castaneda S.F., Gallo L.C., Talavera G.A., Sotres-Alvarez D., Van Horn L., Beasley J.M. (2019). DASH diet and prevalent metabolic syndrome in the Hispanic Community Health Study/Study of Latinos. Prev. Med. Rep..

[23] Ghorabi S., Salari-Moghaddam A., Daneshzad E., Sadeghi O., Azadbakht L., Djafarian K. (2019). Association between the DASH diet and metabolic syndrome components in Iranian adults. Diabetes Metab. Syndr..

[24] Reynolds S.L., Hagedorn A., Yeom J., Saito Y., Yokoyama E., Crimmins E.M. (2008). A tale of two countries—The United States and Japan: Are differences in health due to differences in overweight?. J. Epidemiol..

[25] Wainwright P., Byrne C.D. (2016). Bidirectional Relationships and Disconnects between NAFLD and Features of the Metabolic Syndrome. Int. J. Mol. Sci..

[26] Lonardo A., Nascimbeni F., Mantovani A., Targher G. (2018). Hypertension, diabetes, atherosclerosis and NASH: Cause or consequence?. J. Hepatol..

[27] van der Veen J.N., Kennelly J.P., Wan S., Vance J.E., Vance D.E., Jacobs R.L. (2017). The critical role of phosphatidylcholine and phosphatidylethanolamine metabolism in health and disease. Biochim. Biophys. Acta Biomembr..

[28] Ren J., Pulakat L., Whaley-Connell A., Sowers J.R. (2010). Mitochondrial biogenesis in the metabolic syndrome and cardiovascular disease. J. Mol. Med. (Berlin).

[29] Supale S., Li N., Brun T., Maechler P. (2012). Mitochondrial dysfunction in pancreatic beta cells. Trends Endocrinol. Metab..

[30] Feldman A., Eder S.K., Felder T.K., Kedenko L., Paulweber B., Stadlmayr A., Huber-Schonauer U., Niederseer D., Stickel F., Auer S. (2017). Clinical and Metabolic Characterization of Lean Caucasian Subjects With Non-alcoholic Fatty Liver. Am. J. Gastroenterol..

[31] Allam-Ndoul B., Guenard F., Garneau V., Cormier H., Barbier O., Perusse L., Vohl M.C. (2016). Association between Metabolite Profiles, Metabolic Syndrome and Obesity Status. Nutrients.

[32] Lo C.J., Tang H.Y., Huang C.Y., Lin C.M., Ho H.Y., Shiao M.S., Cheng M.L. (2018). Metabolic Signature Differentiated Diabetes Mellitus from Lipid Disorder in Elderly Taiwanese. J. Clin. Med..

[33] Yang S.J., Kwak S.Y., Jo G., Song T.J., Shin M.J. (2018). Serum metabolite profile associated with incident type 2 diabetes in Koreans: Findings from the Korean Genome and Epidemiology Study. Sci. Rep..

[34] Varma V.R., Oommen A.M., Varma S., Casanova R., An Y., Andrews R.M., O’Brien R., Pletnikova O., Troncoso J.C., Toledo J. (2018). Brain and blood metabolite signatures of pathology and progression in Alzheimer disease: A targeted metabolomics study. PLoS Med..

[35] Brusselmans K., De Schrijver E., Verhoeven G., Swinnen J.V. (2005). RNA interference-mediated silencing of the acetyl-CoA-carboxylase-alpha gene induces growth inhibition and apoptosis of prostate cancer cells. Cancer Res..

[36] Cowart L.A., Cowart L.A. (2011). Sphingolipids and Metabolic Disease.

[37] Hanamatsu H., Ohnishi S., Sakai S., Yuyama K., Mitsutake S., Takeda H., Hashino S., Igarashi Y. (2014). Altered levels of serum sphingomyelin and ceramide containing distinct acyl chains in young obese adults. Nutr. Diabetes.

[38] Papandreou C., Bullo M., Ruiz-Canela M., Dennis C., Deik A., Wang D., Guasch-Ferre M., Yu E., Razquin C., Corella D. (2019). Plasma metabolites predict both insulin resistance and incident type 2 diabetes: A metabolomics approach within the Prevencion con Dieta Mediterranea (PREDIMED) study. Am. J. Clin. Nutr..

[39] Toledo J.B., Arnold M., Kastenmuller G., Chang R., Baillie R.A., Han X., Thambisetty M., Tenenbaum J.D., Suhre K., Thompson J.W. (2017). Metabolic network failures in Alzheimer’s disease: A biochemical road map. Alzheimers Dement..

[40] Li D., Misialek J.R., Boerwinkle E., Gottesman R.F., Sharrett A.R., Mosley T.H., Coresh J., Wruck L.M., Knopman D.S., Alonso A. (2017). Prospective associations of plasma phospholipids and mild cognitive impairment/dementia among African Americans in the ARIC Neurocognitive Study. Alzheimers Dement. (Amsterdam).

[41] Mathews A.T., Famodu O.A., Olfert M.D., Murray P.J., Cuff C.F., Downes M.T., Haughey N.J., Colby S.E., Chantler P.D., Olfert I.M. (2017). Efficacy of nutritional interventions to lower circulating ceramides in young adults: FRUVEDomic pilot study. Physiol. Rep..

[42] Knebel B., Strassburger K., Szendroedi J., Kotzka J., Scheer M., Nowotny B., Mussig K., Lehr S., Pacini G., Finner H. (2016). Specific Metabolic Profiles and Their Relationship to Insulin Resistance in Recent-Onset Type 1 and Type 2 Diabetes. J. Clin. Endocrinol. Metab..

[43] Kamaura M., Nishijima K., Takahashi M., Ando T., Mizushima S., Tochikubo O. (2010). Lifestyle modification in metabolic syndrome and associated changes in plasma amino acid profiles. Circ. J..

[44] Nakamura H., Jinzu H., Nagao K., Noguchi Y., Shimba N., Miyano H., Watanabe T., Iseki K. (2014). Plasma amino acid profiles are associated with insulin, C-peptide and adiponectin levels in type 2 diabetic patients. Nutr. Diabetes.

[45] Newgard C.B., An J., Bain J.R., Muehlbauer M.J., Stevens R.D., Lien L.F., Haqq A.M., Shah S.H., Arlotto M., Slentz C.A. (2009). A branched-chain amino acid-related metabolic signature that differentiates obese and lean humans and contributes to insulin resistance. Cell Metab..

[46] Wang T.J., Larson M.G., Vasan R.S., Cheng S., Rhee E.P., McCabe E., Lewis G.D., Fox C.S., Jacques P.F., Fernandez C. (2011). Metabolite profiles and the risk of developing diabetes. Nat. Med..

[47] Zhao X., Han Q., Liu Y., Sun C., Gang X., Wang G. (2016). The Relationship between Branched-Chain Amino Acid Related Metabolomic Signature and Insulin Resistance: A Systematic Review. J. Diabetes Res..

[48] Takashina C., Tsujino I., Watanabe T., Sakaue S., Ikeda D., Yamada A., Sato T., Ohira H., Otsuka Y., Oyama-Manabe N. (2016). Associations among the plasma amino acid profile, obesity, and glucose metabolism in Japanese adults with normal glucose tolerance. Nutr. Metab. (London).

[49] Iida M., Harada S., Kurihara A., Fukai K., Kuwabara K., Sugiyama D., Takeuchi A., Okamura T., Akiyama M., Nishiwaki Y. (2016). Profiling of plasma metabolites in postmenopausal women with metabolic syndrome. Menopause.

[50] Ruiz-Canela M., Toledo E., Clish C.B., Hruby A., Liang L., Salas-Salvado J., Razquin C., Corella D., Estruch R., Ros E. (2016). Plasma Branched-Chain Amino Acids and Incident Cardiovascular Disease in the PREDIMED Trial. Clin. Chem..

[51] Wang T.J., Ngo D., Psychogios N., Dejam A., Larson M.G., Vasan R.S., Ghorbani A., O’Sullivan J., Cheng S., Rhee E.P. (2013). 2-Aminoadipic acid is a biomarker for diabetes risk. J. Clin. Investig..

[52] Ferrucci L. (2008). The Baltimore Longitudinal Study of Aging (BLSA): A 50-year-long journey and plans for the future. J. Gerontol. A Biol. Sci. Med. Sci..

[53] Resnick S.M., Pham D.L., Kraut M.A., Zonderman A.B., Davatzikos C. (2003). Longitudinal magnetic resonance imaging studies of older adults: A shrinking brain. J. Neurosci..

[54] Harada S., Takebayashi T., Kurihara A., Akiyama M., Suzuki A., Hatakeyama Y., Sugiyama D., Kuwabara K., Takeuchi A., Okamura T. (2016). Metabolomic profiling reveals novel biomarkers of alcohol intake and alcohol-induced liver injury in community-dwelling men. Environ. Health Prev. Med..

[55] Casanova R., Varma S., Simpson B., Kim M., An Y., Saldana S., Riveros C., Moscato P., Griswold M., Sonntag D. (2016). Blood metabolite markers of preclinical Alzheimer’s disease in two longitudinally followed cohorts of older individuals. Alzheimers Dement..

[56] Mellen P.B., Gao S.K., Vitolins M.Z., Goff D.C. (2008). Deteriorating dietary habits among adults with hypertension: DASH dietary accordance, NHANES 1988-1994 and 1999-2004. Arch. Intern. Med..

[57] Murali A.R., Carey W.D. Liver Test Interpretation—Approach to the Patient with Liver Disease: A Guide to Commonly Used Liver Tests. http://www.clevelandclinicmeded.com/medicalpubs/diseasemanagement/hepatology/guide-to-common-liver-tests/.

[58] Cortez-Pinto H., Camilo M.E., Baptista A., De Oliveira A.G., De Moura M.C. (1999). Non-alcoholic fatty liver: Another feature of the metabolic syndrome?. Clin. Nutr..

[59] Souza M.R., Diniz Mde F., Medeiros-Filho J.E., Araujo M.S. (2012). Metabolic syndrome and risk factors for non-alcoholic fatty liver disease. Arq. Gastroenterol..

[60] Verbrugge L.M., Gruber-Baldini A.L., Fozard J.L. (1996). Age differences and age changes in activities: Baltimore Longitudinal Study of Aging. J. Gerontol. B Psychol. Sci. Soc. Sci..

[61] Fujii H., Yamamoto S., Takeda-Imai F., Inoue M., Tsugane S., Kadowaki T., Noda M. (2011). Validity and applicability of a simple questionnaire for the estimation of total and domain-specific physical activity. Diabetol. Int..

[62] Benjamini Y., Hochberg Y. (1995). Controlling the False Discovery Rate: A Practical and Powerful Approach to Multiple Testing. J. R. Stat. Soc. Ser. B (Methodol.).

