# Supplementary material for: Blood Metabolite Signatures of Metabolic Syndrome in Two Cross-Cultural Older Adult Cohorts
Source: Int J Mol Sci. 2020 Feb 16;21(4):1324. doi: 10.3390/ijms21041324 (PMC7072935; doi:10.3390/ijms21041324)
Supplement: Supplementary file 1 [file ijms-21-01324-s001.zip › Supp/Supplementary Text 2_15_20.docx]

Supp. Table S1. Differences in metabolite concentrations between BLSA and TMCS

| Symbol | Full Name | Category | NI (n=146) | JP (n=104) | p-value | FDR adjusted p-value |
| --- | --- | --- | --- | --- | --- | --- |
|  |  |  | Mean (SD) | Mean (SD) |  |  |
| Ala | Alanine | Amino acids | 462.4 (93.7512) | 364.8 (81.8831) | <.0001 | <.0001 |
| Arg | Arginine | Amino acids | 115.1 (21.9527) | 135.6 (22.8932) | <.0001 | <.0001 |
| Asn | Asparagine | Amino acids | 52.7952 (6.9825) | 53.6567 (6.9907) | 0.3381 | 0.37371 |
| Asp | Aspartate | Amino acids | 41.8414 (8.4668) | 22.7625 (6.2785) | <.0001 | <.0001 |
| Cit | Citrulline | Amino acids | 39.2212 (8.263) | 34.1923 (7.8986) | <.0001 | <.0001 |
| Gln | Glutamine | Amino acids | 676.9 (77.6657) | 752.4 (98.1796) | <.0001 | <.0001 |
| Glu | Glutamate | Amino acids | 142.4 (36.2265) | 59.7058 (19.173) | <.0001 | <.0001 |
| Gly | Glycine | Amino acids | 405.5 (83.9716) | 259.7 (53.6299) | <.0001 | <.0001 |
| His | Histidine | Amino acids | 97.2993 (10.9317) | 97.025 (11.2187) | 0.8468 | 0.88911 |
| Ile | Isoleucine | Amino acids | 87.5616 (16.6771) | 77.9587 (17.8873) | <.0001 | 0.00003 |
| Leu | Leucine | Amino acids | 162.5 (21.9765) | 150.1 (31.4236) | 0.0007 | 0.00086 |
| Lys | Lysine | Amino acids | 208.7 (28.478) | 220.5 (34.0519) | 0.0044 | 0.0054 |
| Met | Methionine | Amino acids | 29.9992 (5.0739) | 27.1596 (5.2376) | <.0001 | 0.00003 |
| Orn | Ornithine | Amino acids | 139.8 (24.3316) | 85.9587 (20.1287) | <.0001 | <.0001 |
| Phe | Phenylalanine | Amino acids | 103.1 (13.9991) | 73.9941 (10.2206) | <.0001 | <.0001 |
| Pro | Proline | Amino acids | 218.5 (55.0666) | 130.9 (42.1702) | <.0001 | <.0001 |
| Ser | Serine | Amino acids | 167.2 (25.8559) | 147.7 (21.7614) | <.0001 | <.0001 |
| Thr | Threonine | Amino acids | 136.7 (27.0453) | 131.7 (25.1541) | 0.1364 | 0.15914 |
| Trp | Tryptophan | Amino acids | 64.1 (9.2413) | 64.2019 (10.2785) | 0.9347 | 0.9347 |
| Tyr | Tyrosine | Amino acids | 82.961 (15.0703) | 71.8606 (12.8527) | <.0001 | <.0001 |
| Val | Valine | Amino acids | 297.2 (48.4833) | 245.4 (49.5631) | <.0001 | <.0001 |
| ADMA | Asymmetric dimethylarginine | Biogenic amines | 0.5044 (0.0779) | 0.4896 (0.073) | 0.1303 | 0.18621 |
| alpha-AAA | alpha-Aminoadipic acid | Biogenic amines | 0.9143 (0.3501) | 1.2398 (0.3493) | <.0001 | <.0001 |
| Creatinine | Creatinine | Biogenic amines | 69.5637 (21.9174) | 69.1942 (16.1567) | 0.8782 | 0.8782 |
| Kyn | Kynurenine | Biogenic amines | 2.1901 (0.5232) | 1.9045 (0.459) | <.0001 | 0.00002 |
| Met-SO | Methionine-Sulfoxide | Biogenic amines | 0.9948 (0.4201) | 0.3392 (0.2468) | <.0001 | <.0001 |
| Sarcosine | Sarcosine | Biogenic amines | 1.3486 (0.5232) | 1.7374 (0.7521) | <.0001 | 0.00002 |
| SDMA | Symmetric dimethylarginine | Biogenic amines | 0.6179 (0.1303) | 0.6 (0.1242) | 0.2755 | 0.3444 |
| Serotonin | Serotonin | Biogenic amines | 0.469 (0.2518) | 0.4944 (0.2215) | 0.4098 | 0.45534 |
| t4-OH-Pro | trans-4-Hydroxyproline | Biogenic amines | 12.8162 (5.4865) | 8.2084 (2.6927) | <.0001 | <.0001 |
| Taurine | Taurine | Biogenic amines | 170 (32.4623) | 126.8 (24.7324) | <.0001 | <.0001 |
| C0 | Carnitine (free) | acylcarnitines | 37.4911 (6.8287) | 43.9933 (8.413) | <.0001 | <.0001 |
| C2 | Acetylcarnitine | acylcarnitines | 6.3925 (1.8963) | 8.4791 (2.4032) | <.0001 | <.0001 |
| C3 | Propionylcarnitine | acylcarnitines | 0.3248 (0.0889) | 0.3892 (0.1226) | <.0001 | 0.00001 |
| C4 | Butyrylcarnitine / Isobutyrylcarnitine | acylcarnitines | 0.2608 (0.0926) | 0.1995 (0.0492) | <.0001 | <.0001 |
| C5 | Isovalerylcarnitine / 2-Methylbutyrylcarnitine / Valerylcarnitine | acylcarnitines | 0.1647 (0.0521) | 0.128 (0.0336) | <.0001 | <.0001 |
| C10 | Decanoylcarnitine [= Caprylcarnitine] | acylcarnitines | 0.2567 (0.1087) | 0.3544 (0.1148) | <.0001 | <.0001 |
| C14:1 | Tetradecenoylcarnitine [= Myristoleylcarnitine] | acylcarnitines | 0.0928 (0.0275) | 0.1623 (0.0507) | <.0001 | <.0001 |
| C14:2 | Tetradecadienoylcarnitine | acylcarnitines | 0.0339 (0.0147) | 0.0669 (0.0257) | <.0001 | <.0001 |
| C16 | Hexadecanoylcarnitine [= Palmitoylcarnitine] | acylcarnitines | 0.0798 (0.0177) | 0.1302 (0.0253) | <.0001 | <.0001 |
| C18 | Octadecanoylcarnitine [= Stearylcarnitine] | acylcarnitines | 2.1667 (0.5485) | 0.0476 (0.0118) | <.0001 | <.0001 |
| C18:1 | Octadecenoylcarnitine [= Oleylcarnitine] | acylcarnitines | 0.168 (0.0381) | 0.1613 (0.0339) | 0.154 | 0.16801 |
| C18:2 | Octadecadienoylcarnitine [= Linoleylcarnitine] | acylcarnitines | 0.0875 (0.0217) | 0.0906 (0.0206) | 0.2529 | 0.2529 |
| H1 | Glucose | hexoses | 6159.6 (1023.8) | 5366 (735.7) | <.0001 | <.0001 |
| lysoPC a C16:0 | Lysophosphatidylcholine with acyl residue C16:0 | Lysophosphatidylcholines | 152.3 (25.5871) | 88.8519 (15.1025) | <.0001 | <.0001 |
| lysoPC a C16:1 | Lysophosphatidylcholine with acyl residue C16:1 | Lysophosphatidylcholines | 3.7384 (0.9274) | 2.4824 (0.6056) | <.0001 | <.0001 |
| lysoPC a C17:0 | Lysophosphatidylcholine with acyl residue C17:0 | Lysophosphatidylcholines | 2.7375 (0.5402) | 1.4976 (0.3412) | <.0001 | <.0001 |
| lysoPC a C18:0 | Lysophosphatidylcholine with acyl residue C18:0 | Lysophosphatidylcholines | 30.7363 (5.4766) | 28.8625 (5.4438) | 0.008 | 0.00882 |
| lysoPC a C18:1 | Lysophosphatidylcholine with acyl residue C18:1 | Lysophosphatidylcholines | 21.7397 (4.8872) | 16.2133 (3.9836) | <.0001 | <.0001 |
| lysoPC a C18:2 | Lysophosphatidylcholine with acyl residue C18:2 | Lysophosphatidylcholines | 26.7747 (7.6489) | 20.6356 (6.0285) | <.0001 | <.0001 |
| lysoPC a C20:3 | Lysophosphatidylcholine with acyl residue C20:3 | Lysophosphatidylcholines | 1.6736 (0.4931) | 1.144 (0.4188) | <.0001 | <.0001 |
| lysoPC a C20:4 | Lysophosphatidylcholine with acyl residue C20:4 | Lysophosphatidylcholines | 7.5509 (2.3929) | 3.8938 (1.1816) | <.0001 | <.0001 |
| lysoPC a C26:0 | Lysophosphatidylcholine with acyl residue C26:0 | Lysophosphatidylcholines | 0.2695 (0.1704) | 0.2428 (0.1158) | 0.1466 | 0.14661 |
| lysoPC a C28:0 | Lysophosphatidylcholine with acyl residue C28:0 | Lysophosphatidylcholines | 0.2548 (0.1097) | 0.1732 (0.0706) | <.0001 | <.0001 |
| lysoPC a C28:1 | Lysophosphatidylcholine with acyl residue C28:1 | Lysophosphatidylcholines | 0.3916 (0.2149) | 0.2768 (0.0853) | <.0001 | <.0001 |
| PC aa C28:1 | Phosphatidylcholine with diacyl residue sum C28:1 | Phosphatidylcholines-diacyl | 2.9273 (0.8214) | 2.6119 (0.6347) | 0.0007 | 0.00084 |
| PC aa C30:0 | Phosphatidylcholine with diacyl residue sum C30:0 | Phosphatidylcholines-diacyl | 2.43 (0.761) | 3.5513 (1.054) | <.0001 | <.0001 |
| PC aa C32:0 | Phosphatidylcholine with diacyl residue sum C32:0 | Phosphatidylcholines-diacyl | 9.7382 (2.1177) | 15.2133 (3.3129) | <.0001 | <.0001 |
| PC aa C32:1 | Phosphatidylcholine with diacyl residue sum C32:1 | Phosphatidylcholines-diacyl | 8.2929 (3.64) | 16.4083 (7.2552) | <.0001 | <.0001 |
| PC aa C32:2 | Phosphatidylcholine with diacyl residue sum C32:2 | Phosphatidylcholines-diacyl | 2.5521 (1.129) | 3.548 (1.0943) | <.0001 | <.0001 |
| PC aa C32:3 | Phosphatidylcholine with diacyl residue sum C32:3 | Phosphatidylcholines-diacyl | 0.4177 (0.1087) | 0.4958 (0.1032) | <.0001 | <.0001 |
| PC aa C34:1 | Phosphatidylcholine with diacyl residue sum C34:1 | Phosphatidylcholines-diacyl | 135.7 (31.5878) | 206.4 (42.6005) | <.0001 | <.0001 |
| PC aa C34:2 | Phosphatidylcholine with diacyl residue sum C34:2 | Phosphatidylcholines-diacyl | 275 (44.4406) | 343.3 (62.4736) | <.0001 | <.0001 |
| PC aa C34:3 | Phosphatidylcholine with diacyl residue sum C34:3 | Phosphatidylcholines-diacyl | 12.4382 (3.5296) | 13.9278 (3.4603) | 0.0011 | 0.00119 |
| PC aa C34:4 | Phosphatidylcholine with diacyl residue sum C34:4 | Phosphatidylcholines-diacyl | 1.5121 (0.5747) | 1.3139 (0.4066) | 0.0016 | 0.00172 |
| PC aa C36:0 | Phosphatidylcholine with diacyl residue sum C36:0 | Phosphatidylcholines-diacyl | 3.2621 (0.9988) | 6.1938 (2.4003) | <.0001 | <.0001 |
| PC aa C36:1 | Phosphatidylcholine with diacyl residue sum C36:1 | Phosphatidylcholines-diacyl | 28.9747 (6.9173) | 45.568 (9.9016) | <.0001 | <.0001 |
| PC aa C36:2 | Phosphatidylcholine with diacyl residue sum C36:2 | Phosphatidylcholines-diacyl | 155.4 (29.8499) | 210.3 (39.8486) | <.0001 | <.0001 |
| PC aa C36:3 | Phosphatidylcholine with diacyl residue sum C36:3 | Phosphatidylcholines-diacyl | 83.3938 (17.0607) | 98.2875 (23.8871) | <.0001 | <.0001 |
| PC aa C36:4 | Phosphatidylcholine with diacyl residue sum C36:4 | Phosphatidylcholines-diacyl | 132.9 (29.277) | 149.5 (36.0612) | 0.0002 | 0.00018 |
| PC aa C36:5 | Phosphatidylcholine with diacyl residue sum C36:5 | Phosphatidylcholines-diacyl | 38.9873 (19.8867) | 75.025 (34.1588) | <.0001 | <.0001 |
| PC aa C36:6 | Phosphatidylcholine with diacyl residue sum C36:6 | Phosphatidylcholines-diacyl | 1.6697 (0.7125) | 2.0041 (0.7316) | 0.0004 | 0.00046 |
| PC aa C38:0 | Phosphatidylcholine with diacyl residue sum C38:0 | Phosphatidylcholines-diacyl | 3.7247 (1.0237) | 4.6846 (1.2289) | <.0001 | <.0001 |
| PC aa C38:1 | Phosphatidylcholine with diacyl residue sum C38:1 | Phosphatidylcholines-diacyl | 1.202 (0.3877) | 1.7655 (0.6086) | <.0001 | <.0001 |
| PC aa C38:3 | Phosphatidylcholine with diacyl residue sum C38:3 | Phosphatidylcholines-diacyl | 29.9767 (7.4669) | 37.8413 (9.1773) | <.0001 | <.0001 |
| PC aa C38:4 | Phosphatidylcholine with diacyl residue sum C38:4 | Phosphatidylcholines-diacyl | 82.3164 (21.7914) | 82.1827 (22.2472) | 0.9622 | 0.96222 |
| PC aa C38:5 | Phosphatidylcholine with diacyl residue sum C38:5 | Phosphatidylcholines-diacyl | 44.4411 (11.4912) | 79.7731 (23.4877) | <.0001 | <.0001 |
| PC aa C38:6 | Phosphatidylcholine with diacyl residue sum C38:6 | Phosphatidylcholines-diacyl | 93.9719 (25.3949) | 149 (32.0215) | <.0001 | <.0001 |
| PC aa C40:2 | Phosphatidylcholine with diacyl residue sum C40:2 | Phosphatidylcholines-diacyl | 0.6003 (0.1426) | 0.4478 (0.1375) | <.0001 | <.0001 |
| PC aa C40:3 | Phosphatidylcholine with diacyl residue sum C40:3 | Phosphatidylcholines-diacyl | 0.5913 (0.1202) | 0.7473 (0.1939) | <.0001 | <.0001 |
| PC aa C40:4 | Phosphatidylcholine with diacyl residue sum C40:4 | Phosphatidylcholines-diacyl | 2.4855 (0.7224) | 2.2492 (0.5308) | 0.0032 | 0.00333 |
| PC aa C40:5 | Phosphatidylcholine with diacyl residue sum C40:5 | Phosphatidylcholines-diacyl | 7.6736 (2.1238) | 12.2248 (3.8282) | <.0001 | <.0001 |
| PC aa C40:6 | Phosphatidylcholine with diacyl residue sum C40:6 | Phosphatidylcholines-diacyl | 34.9377 (10.2389) | 56.1644 (14.9888) | <.0001 | <.0001 |
| PC aa C42:0 | Phosphatidylcholine with diacyl residue sum C42:0 | Phosphatidylcholines-diacyl | 0.5613 (0.1608) | 0.8575 (0.2506) | <.0001 | <.0001 |
| PC aa C42:1 | Phosphatidylcholine with diacyl residue sum C42:1 | Phosphatidylcholines-diacyl | 0.3249 (0.0952) | 0.4648 (0.1185) | <.0001 | <.0001 |
| PC aa C42:2 | Phosphatidylcholine with diacyl residue sum C42:2 | Phosphatidylcholines-diacyl | 0.2504 (0.0737) | 0.45 (0.1248) | <.0001 | <.0001 |
| PC aa C42:4 | Phosphatidylcholine with diacyl residue sum C42:4 | Phosphatidylcholines-diacyl | 0.2275 (0.0495) | 0.1899 (0.0401) | <.0001 | <.0001 |
| PC aa C42:5 | Phosphatidylcholine with diacyl residue sum C42:5 | Phosphatidylcholines-diacyl | 0.2948 (0.0732) | 0.5211 (0.1536) | <.0001 | <.0001 |
| PC aa C42:6 | Phosphatidylcholine with diacyl residue sum C42:6 | Phosphatidylcholines-diacyl | 0.3602 (0.089) | 0.8038 (0.2431) | <.0001 | <.0001 |
| PC ae C30:0 | Phosphatidylcholine with acyl-alkyl residue sum C30:0 | Phosphatidylcholines-acyl-alkyl | 0.218 (0.0649) | 0.2962 (0.0786) | <.0001 | <.0001 |
| PC ae C30:1 | Phosphatidylcholine with acyl-alkyl residue sum C30:1 | Phosphatidylcholines-acyl-alkyl | 0.1217 (0.0919) | 0.1055 (0.0665) | 0.107 | 0.12838 |
| PC ae C32:1 | Phosphatidylcholine with acyl-alkyl residue sum C32:1 | Phosphatidylcholines-acyl-alkyl | 1.5974 (0.3525) | 2.5918 (0.5602) | <.0001 | <.0001 |
| PC ae C32:2 | Phosphatidylcholine with acyl-alkyl residue sum C32:2 | Phosphatidylcholines-acyl-alkyl | 0.5119 (0.1295) | 0.8518 (0.1973) | <.0001 | <.0001 |
| PC ae C34:0 | Phosphatidylcholine with acyl-alkyl residue sum C34:0 | Phosphatidylcholines-acyl-alkyl | 0.9012 (0.224) | 1.5964 (0.4695) | <.0001 | <.0001 |
| PC ae C34:1 | Phosphatidylcholine with acyl-alkyl residue sum C34:1 | Phosphatidylcholines-acyl-alkyl | 5.4713 (1.1767) | 8.1523 (1.4995) | <.0001 | <.0001 |
| PC ae C34:2 | Phosphatidylcholine with acyl-alkyl residue sum C34:2 | Phosphatidylcholines-acyl-alkyl | 7.1326 (1.8395) | 9.9801 (2.3837) | <.0001 | <.0001 |
| PC ae C34:3 | Phosphatidylcholine with acyl-alkyl residue sum C34:3 | Phosphatidylcholines-acyl-alkyl | 4.9816 (1.5497) | 7.2291 (2.1582) | <.0001 | <.0001 |
| PC ae C36:0 | Phosphatidylcholine with acyl-alkyl residue sum C36:0 | Phosphatidylcholines-acyl-alkyl | 0.7722 (0.1751) | 1.3258 (0.3777) | <.0001 | <.0001 |
| PC ae C36:1 | Phosphatidylcholine with acyl-alkyl residue sum C36:1 | Phosphatidylcholines-acyl-alkyl | 9.0631 (1.9585) | 6.8306 (1.3284) | <.0001 | <.0001 |
| PC ae C36:2 | Phosphatidylcholine with acyl-alkyl residue sum C36:2 | Phosphatidylcholines-acyl-alkyl | 10.2562 (2.2398) | 11.2598 (2.8718) | 0.0033 | 0.00424 |
| PC ae C36:3 | Phosphatidylcholine with acyl-alkyl residue sum C36:3 | Phosphatidylcholines-acyl-alkyl | 5.2947 (1.3417) | 5.9738 (1.3798) | 0.0001 | 0.00019 |
| PC ae C36:4 | Phosphatidylcholine with acyl-alkyl residue sum C36:4 | Phosphatidylcholines-acyl-alkyl | 12.2834 (2.8929) | 12.492 (3.1664) | 0.5894 | 0.64303 |
| PC ae C36:5 | Phosphatidylcholine with acyl-alkyl residue sum C36:5 | Phosphatidylcholines-acyl-alkyl | 9.2967 (2.3476) | 12.1723 (3.1392) | <.0001 | <.0001 |
| PC ae C38:0 | Phosphatidylcholine with acyl-alkyl residue sum C38:0 | Phosphatidylcholines-acyl-alkyl | 3.4338 (1.1353) | 3.6712 (1.0087) | 0.0911 | 0.11303 |
| PC ae C38:1 | Phosphatidylcholine with acyl-alkyl residue sum C38:1 | Phosphatidylcholines-acyl-alkyl | 1.5016 (0.5959) | 0.8033 (0.2477) | <.0001 | <.0001 |
| PC ae C38:2 | Phosphatidylcholine with acyl-alkyl residue sum C38:2 | Phosphatidylcholines-acyl-alkyl | 3.2823 (0.7488) | 1.9919 (0.4356) | <.0001 | <.0001 |
| PC ae C38:3 | Phosphatidylcholine with acyl-alkyl residue sum C38:3 | Phosphatidylcholines-acyl-alkyl | 6.3184 (1.3543) | 3.0613 (0.6187) | <.0001 | <.0001 |
| PC ae C38:4 | Phosphatidylcholine with acyl-alkyl residue sum C38:4 | Phosphatidylcholines-acyl-alkyl | 9.646 (1.9859) | 7.6338 (1.9387) | <.0001 | <.0001 |
| PC ae C38:5 | Phosphatidylcholine with acyl-alkyl residue sum C38:5 | Phosphatidylcholines-acyl-alkyl | 13.7958 (2.6743) | 13.6626 (3.2563) | 0.732 | 0.7751 |
| PC ae C38:6 | Phosphatidylcholine with acyl-alkyl residue sum C38:6 | Phosphatidylcholines-acyl-alkyl | 7.4868 (2.0171) | 10.2861 (2.5678) | <.0001 | <.0001 |
| PC ae C40:1 | Phosphatidylcholine with acyl-alkyl residue sum C40:1 | Phosphatidylcholines-acyl-alkyl | 1.474 (0.3705) | 1.5483 (0.3641) | 0.1169 | 0.13578 |
| PC ae C40:2 | Phosphatidylcholine with acyl-alkyl residue sum C40:2 | Phosphatidylcholines-acyl-alkyl | 2.0361 (0.4809) | 2.0248 (0.466) | 0.8528 | 0.85281 |
| PC ae C40:3 | Phosphatidylcholine with acyl-alkyl residue sum C40:3 | Phosphatidylcholines-acyl-alkyl | 2.287 (0.4569) | 0.9877 (0.1868) | <.0001 | <.0001 |
| PC ae C40:4 | Phosphatidylcholine with acyl-alkyl residue sum C40:4 | Phosphatidylcholines-acyl-alkyl | 2.497 (0.5158) | 1.6494 (0.3719) | <.0001 | <.0001 |
| PC ae C40:5 | Phosphatidylcholine with acyl-alkyl residue sum C40:5 | Phosphatidylcholines-acyl-alkyl | 4.7359 (0.9127) | 3.7184 (0.9338) | <.0001 | <.0001 |
| PC ae C40:6 | Phosphatidylcholine with acyl-alkyl residue sum C40:6 | Phosphatidylcholines-acyl-alkyl | 5.0267 (1.1723) | 6.1493 (1.5517) | <.0001 | <.0001 |
| PC ae C42:1 | Phosphatidylcholine with acyl-alkyl residue sum C42:1 | Phosphatidylcholines-acyl-alkyl | 0.3645 (0.0859) | 0.3767 (0.0782) | 0.2511 | 0.2825 |
| PC ae C42:2 | Phosphatidylcholine with acyl-alkyl residue sum C42:2 | Phosphatidylcholines-acyl-alkyl | 0.5317 (0.1197) | 0.6208 (0.1333) | <.0001 | <.0001 |
| PC ae C42:3 | Phosphatidylcholine with acyl-alkyl residue sum C42:3 | Phosphatidylcholines-acyl-alkyl | 0.8596 (0.1891) | 0.8647 (0.1829) | 0.8318 | 0.85281 |
| PC ae C42:4 | Phosphatidylcholine with acyl-alkyl residue sum C42:4 | Phosphatidylcholines-acyl-alkyl | 0.8121 (0.1998) | 0.6399 (0.1985) | <.0001 | <.0001 |
| PC ae C42:5 | Phosphatidylcholine with acyl-alkyl residue sum C42:5 | Phosphatidylcholines-acyl-alkyl | 2.3212 (0.4669) | 1.5121 (0.3743) | <.0001 | <.0001 |
| PC ae C44:3 | Phosphatidylcholine with acyl-alkyl residue sum C44:3 | Phosphatidylcholines-acyl-alkyl | 0.1334 (0.0321) | 0.192 (0.0417) | <.0001 | <.0001 |
| PC ae C44:4 | Phosphatidylcholine with acyl-alkyl residue sum C44:4 | Phosphatidylcholines-acyl-alkyl | 0.3137 (0.0718) | 0.2866 (0.0684) | 0.0031 | 0.0041 |
| PC ae C44:5 | Phosphatidylcholine with acyl-alkyl residue sum C44:5 | Phosphatidylcholines-acyl-alkyl | 1.2494 (0.3442) | 1.116 (0.3378) | 0.0026 | 0.00358 |
| PC ae C44:6 | Phosphatidylcholine with acyl-alkyl residue sum C44:6 | Phosphatidylcholines-acyl-alkyl | 0.9751 (0.25) | 1.1729 (0.3519) | <.0001 | <.0001 |
| SM (OH) C14:1 | Hydroxysphingomyelin with acyl residue sum C14:1 | Sphingomyelins | 6.0218 (1.4917) | 4.7205 (1.2521) | <.0001 | <.0001 |
| SM (OH) C16:1 | Hydroxysphingomyelin with acyl residue sum C16:1 | Sphingomyelins | 3.0439 (0.6946) | 2.6972 (0.6645) | <.0001 | 0.00014 |
| SM (OH) C22:1 | Hydroxysphingomyelin with acyl residue sum C22:1 | Sphingomyelins | 13.4668 (2.803) | 9.94 (2.3016) | <.0001 | <.0001 |
| SM (OH) C22:2 | Hydroxysphingomyelin with acyl residue sum C22:2 | Sphingomyelins | 9.6649 (2.3427) | 9.6188 (2.2494) | 0.876 | 0.87602 |
| SM (OH) C24:1 | Hydroxysphingomyelin with acyl residue sum C24:1 | Sphingomyelins | 1.2522 (0.2853) | 0.8787 (0.2448) | <.0001 | <.0001 |
| SM C16:0 | Sphingomyelin with acyl residue sum C16:0 | Sphingomyelins | 89.5459 (15.7031) | 112.3 (18.3926) | <.0001 | <.0001 |
| SM C16:1 | Sphingomyelin with acyl residue sum C16:1 | Sphingomyelins | 14.509 (3.0962) | 15.5753 (3.3209) | 0.0098 | 0.01141 |
| SM C18:0 | Sphingomyelin with acyl residue sum C18:0 | Sphingomyelins | 18.1615 (4.3488) | 20.3529 (4.2228) | <.0001 | 0.00014 |
| SM C18:1 | Sphingomyelin with acyl residue sum C18:1 | Sphingomyelins | 9.4561 (2.5084) | 10.5134 (2.8189) | 0.002 | 0.00258 |
| SM C20:2 | Sphingomyelin with acyl residue sum C20:2 | Sphingomyelins | 0.4206 (0.1271) | 0.3051 (0.0925) | <.0001 | <.0001 |
| SM C24:0 | Sphingomyelin with acyl residue sum C24:0 | Sphingomyelins | 19.8694 (4.0155) | 19.2462 (3.5137) | 0.2047 | 0.22048 |
| SM C24:1 | Sphingomyelin with acyl residue sum C24:1 | Sphingomyelins | 41.3959 (8.1836) | 54.2587 (8.9261) | <.0001 | <.0001 |
| SM C26:0 | Sphingomyelin with acyl residue sum C26:0 | Sphingomyelins | 0.1988 (0.0517) | 0.1418 (0.0352) | <.0001 | <.0001 |
| SM C26:1 | Sphingomyelin with acyl residue sum C26:1 | Sphingomyelins | 0.3794 (0.1014) | 0.58 (0.1634) | <.0001 | <.0001 |
| CA | Cholic Acid | Bile Acids | 0.131 (0.1707) | 0.1372 (0.173) | 0.7877 | 0.78771 |
| CDCA | Chenodeoxycholic Acid | Bile Acids | 0.144 (0.1769) | 0.3144 (0.3559) | <.0001 | 0.00005 |
| DCA | Deoxycholic Acid | Bile Acids | 0.4959 (0.3835) | 0.3256 (0.3039) | 0.0002 | 0.0002 |
| GCA | Glycocholic Acid | Bile Acids | 0.209 (0.1688) | 0.0871 (0.0789) | <.0001 | <.0001 |
| GCDCA | Glycochenodeoxycholic Acid | Bile Acids | 0.8238 (0.6801) | 0.3722 (0.3237) | <.0001 | <.0001 |
| GDCA | Glycodeoxycholic Acid | Bile Acids | 0.4145 (0.3226) | 0.1598 (0.1729) | <.0001 | <.0001 |
| GUDCA | Glycoursodeoxycholic Acid | Bile Acids | 0.0705 (0.0636) | 0.0456 (0.0452) | 0.0006 | 0.00065 |
| TCA | Taurocholic Acid | Bile Acids | 0.0366 (0.0432) | 0.0193 (0.0236) | 0.0001 | 0.00016 |
| TCDCA | Taurochenodeoxycholic Acid | Bile Acids | 0.1039 (0.1025) | 0.0452 (0.0423) | <.0001 | <.0001 |
| TDCA | Taurodeoxycholic Acid | Bile Acids | 0.0568 (0.0558) | 0.0175 (0.0206) | <.0001 | <.0001 |
| c-C18:1w9 | Oleic acid | Free Fatty Acid | 314 (92.1129) | 196.5 (55.2239) | <.0001 | <.0001 |
| c-C18:2w6 | Linoleic acid | Free Fatty Acid | 461.8 (96.4932) | 216.3 (83.475) | <.0001 | <.0001 |
| c-C20:1w9 | cis-11-Eicosenoic acid | Free Fatty Acid | 2.9488 (0.9078) | 12.2295 (5.266) | <.0001 | <.0001 |
| c-C20:2w6 | cis-11,14-Eicosadienoic acid | Free Fatty Acid | 5.2996 (1.4434) | 3.4855 (1.4012) | <.0001 | <.0001 |
| c-C20:3w6 | cis-8,11,14-Eicosatrienoic acid | Free Fatty Acid | 26.9813 (9.1961) | 1.9351 (0.7398) | <.0001 | <.0001 |
| c-C20:4w6 | Arachidonic acid | Free Fatty Acid | 187.9 (54.6776) | 8.5835 (2.6077) | <.0001 | <.0001 |
| c-C20:5w3 (EPA) | Eicosapentaenoic acid | Free Fatty Acid | 19.5622 (12.1287) | 4.892 (2.8066) | <.0001 | <.0001 |
| c-C22:6w3 (DHA) | cis-4,7,10,13,16,19-docosahexaenoic acid | Free Fatty Acid | 82.3349 (27.2829) | 29.1753 (11.4721) | <.0001 | <.0001 |
| N-C16:0-Cer |  | Ceramides | 0.2582 (0.0475) | 0.4381 (0.079) | <.0001 | <.0001 |
| N-C18:0-Cer |  | Ceramides | 0.088 (0.0307) | 0.1713 (0.0413) | <.0001 | <.0001 |
| N-C20:0-Cer |  | Ceramides | 0.0983 (0.0275) | 0.1442 (0.0384) | <.0001 | <.0001 |
| N-C22:0-Cer |  | Ceramides | 0.5299 (0.1314) | 0.7519 (0.1886) | <.0001 | <.0001 |
| N-C23:0-Cer |  | Ceramides | 0.781 (0.1831) | 0.8013 (0.2227) | 0.446 | 0.44597 |
| N-C24:0-Cer |  | Ceramides | 2.5607 (0.5765) | 2.3485 (0.6586) | 0.0074 | 0.00826 |
| N-C24:0-Cer(2H) |  | Ceramides | 0.1559 (0.0467) | 0.1947 (0.0789) | <.0001 | 0.00002 |
| N-C24:1-Cer |  | Ceramides | 0.9537 (0.2172) | 1.4827 (0.2939) | <.0001 | <.0001 |
| N-C25:0-Cer |  | Ceramides | 0.1683 (0.0554) | 0.3624 (0.0857) | <.0001 | <.0001 |
| N-C26:1-Cer |  | Ceramides | 0.0133 (0.00877) | 0.0521 (0.023) | <.0001 | <.0001 |

Two-sample t-tests were used to compare age and sex adjusted metabolite values.

BLSA: Baltimore Longitudinal Study of Aging; TMCS: Tsuruoka Metabolomics Cohort Study; SE: standard error.

Lysopcac: Lysophosphatidylcholines. Pcaac: Phosphatidylcholines-diacyl. Pcaec: Phosphatidylcholines-acyl-alkyl. Sm: Sphingomyelins.

Supp. Table S2. Adjusted Significant Associations Unique to BLSA or TMCS

Supp. Table S2a. MetS in BLSA only

| Symbol | Full Name | Category | Odds Ratio Estimate | Lower 95% CI | Upper 95% CI | P value | FDR adjusted p value | Direction of Association |
| --- | --- | --- | --- | --- | --- | --- | --- | --- |
| C2* | Acetylcarnitine | acylcarnitines | 1.861 | 1.205 | 2.875 | 0.0051 | 0.02477 | + |
| C3 | Propionylcarnitine | acylcarnitines | 2.011 | 1.22 | 3.316 | 0.0062 | 0.02477 | + |
| C5* | Isovalerylcarnitine / 2-Methylbutyrylcarnitine / Valerylcarnitine | acylcarnitines | 2.044 | 1.265 | 3.303 | 0.0035 | 0.02477 | + |
| C16 | Hexadecanoylcarnitine [= Palmitoylcarnitine] | acylcarnitines | 1.824 | 1.133 | 2.936 | 0.0134 | 0.04075 | + |
| alpha-AAA* | alpha-Aminoadipic acid | Biogenic amines | 1.89 | 1.275 | 2.801 | 0.0015 | 0.0149 | + |
| N-C24:0-Cer(2H)* |  | Ceramides | 0.466 | 0.285 | 0.762 | 0.0023 | 0.02339 | - |
| N-C25:0-Cer* |  | Ceramides | 0.503 | 0.301 | 0.839 | 0.0085 | 0.04138 | - |
| lysoPC a C18:1* | Lysophosphatidylcholine with acyl residue C18:1 | Lysophosphatidylcholines | 0.502 | 0.307 | 0.819 | 0.0058 | 0.03072 | - |
| lysoPC a C18:2* | Lysophosphatidylcholine with acyl residue C18:2 | Lysophosphatidylcholines | 0.463 | 0.276 | 0.777 | 0.0036 | 0.03072 | - |
| PC ae C30:0* | Phosphatidylcholine with acyl-alkyl residue sum C30:0 | Phosphatidylcholines-acyl-alkyl | 0.336 | 0.191 | 0.591 | 0.0002 | 0.00046 | - |
| PC ae C30:1 | Phosphatidylcholine with acyl-alkyl residue sum C30:1 | Phosphatidylcholines-acyl-alkyl | 0.645 | 0.424 | 0.98 | 0.0398 | 0.04994 | - |
| PC ae C32:1* | Phosphatidylcholine with acyl-alkyl residue sum C32:1 | Phosphatidylcholines-acyl-alkyl | 0.195 | 0.098 | 0.386 | <.0001 | 7.00E-05 | - |
| PC ae C32:2* | Phosphatidylcholine with acyl-alkyl residue sum C32:2 | Phosphatidylcholines-acyl-alkyl | 0.385 | 0.214 | 0.692 | 0.0014 | 0.00267 | - |
| PC ae C34:0* | Phosphatidylcholine with acyl-alkyl residue sum C34:0 | Phosphatidylcholines-acyl-alkyl | 0.594 | 0.382 | 0.925 | 0.0211 | 0.02784 | - |
| PC ae C34:1* | Phosphatidylcholine with acyl-alkyl residue sum C34:1 | Phosphatidylcholines-acyl-alkyl | 0.254 | 0.133 | 0.483 | <.0001 | 0.00016 | - |
| PC ae C36:0* | Phosphatidylcholine with acyl-alkyl residue sum C36:0 | Phosphatidylcholines-acyl-alkyl | 0.505 | 0.318 | 0.805 | 0.004 | 0.00714 | - |
| PC ae C36:1* | Phosphatidylcholine with acyl-alkyl residue sum C36:1 | Phosphatidylcholines-acyl-alkyl | 0.526 | 0.32 | 0.863 | 0.0111 | 0.01573 | - |
| PC ae C36:2* | Phosphatidylcholine with acyl-alkyl residue sum C36:2 | Phosphatidylcholines-acyl-alkyl | 0.21 | 0.106 | 0.418 | <.0001 | 1.00E-04 | - |
| PC ae C38:1* | Phosphatidylcholine with acyl-alkyl residue sum C38:1 | Phosphatidylcholines-acyl-alkyl | 0.395 | 0.23 | 0.678 | 0.0007 | 0.00167 | - |
| PC ae C38:3* | Phosphatidylcholine with acyl-alkyl residue sum C38:3 | Phosphatidylcholines-acyl-alkyl | 0.49 | 0.3 | 0.802 | 0.0045 | 0.00772 | - |
| PC ae C40:1* | Phosphatidylcholine with acyl-alkyl residue sum C40:1 | Phosphatidylcholines-acyl-alkyl | 0.548 | 0.333 | 0.902 | 0.0181 | 0.02421 | - |
| PC ae C40:3* | Phosphatidylcholine with acyl-alkyl residue sum C40:3 | Phosphatidylcholines-acyl-alkyl | 0.283 | 0.151 | 0.529 | <.0001 | 0.00031 | - |
| PC ae C40:4* | Phosphatidylcholine with acyl-alkyl residue sum C40:4 | Phosphatidylcholines-acyl-alkyl | 0.415 | 0.246 | 0.698 | 0.0009 | 0.00191 | - |
| PC ae C40:5* | Phosphatidylcholine with acyl-alkyl residue sum C40:5 | Phosphatidylcholines-acyl-alkyl | 0.539 | 0.341 | 0.852 | 0.0081 | 0.0118 | - |
| PC ae C42:1* | Phosphatidylcholine with acyl-alkyl residue sum C42:1 | Phosphatidylcholines-acyl-alkyl | 0.494 | 0.295 | 0.828 | 0.0075 | 0.01177 | - |
| PC ae c42:2* | Phosphatidylcholine with acyl-alkyl residue sum C42:2 | Phosphatidylcholines-acyl-alkyl | 0.39 | 0.221 | 0.686 | 0.0011 | 0.00216 | - |
| PC ae C42:3* | Phosphatidylcholine with acyl-alkyl residue sum C42:3 | Phosphatidylcholines-acyl-alkyl | 0.297 | 0.16 | 0.551 | 0.0001 | 4.00E-04 | - |
| PC ae C42:4* | Phosphatidylcholine with acyl-alkyl residue sum C42:4 | Phosphatidylcholines-acyl-alkyl | 0.293 | 0.166 | 0.518 | <.0001 | 0.00014 | - |
| PC ae C42:5* | Phosphatidylcholine with acyl-alkyl residue sum C42:5 | Phosphatidylcholines-acyl-alkyl | 0.394 | 0.235 | 0.661 | 0.0004 | 0.00098 | - |
| PC ae C44:3* | Phosphatidylcholine with acyl-alkyl residue sum C44:3 | Phosphatidylcholines-acyl-alkyl | 0.404 | 0.245 | 0.667 | 0.0004 | 0.00098 | - |
| PC ae C44:4* | Phosphatidylcholine with acyl-alkyl residue sum C44:4 | Phosphatidylcholines-acyl-alkyl | 0.352 | 0.206 | 0.6 | 0.0001 | 4.00E-04 | - |
| PC ae C44:5* | Phosphatidylcholine with acyl-alkyl residue sum C44:5 | Phosphatidylcholines-acyl-alkyl | 0.339 | 0.19 | 0.606 | 0.0003 | 7.00E-04 | - |
| PC ae C44:6* | Phosphatidylcholine with acyl-alkyl residue sum C44:6 | Phosphatidylcholines-acyl-alkyl | 0.505 | 0.305 | 0.835 | 0.0078 | 0.0118 | - |
| PC aa C30:0* | Phosphatidylcholine with diacyl residue sum C30:0 | Phosphatidylcholines-diacyl | 0.463 | 0.278 | 0.77 | 0.003 | 0.01446 | - |
| PC aa C32:0* | Phosphatidylcholine with diacyl residue sum C32:0 | Phosphatidylcholines-diacyl | 0.484 | 0.271 | 0.864 | 0.0142 | 0.03595 | - |
| PC aa C32:2* | Phosphatidylcholine with diacyl residue sum C32:2 | Phosphatidylcholines-diacyl | 0.517 | 0.307 | 0.87 | 0.013 | 0.03595 | - |
| PC aa C34:2* | Phosphatidylcholine with diacyl residue sum C34:2 | Phosphatidylcholines-diacyl | 0.44 | 0.259 | 0.748 | 0.0024 | 0.01446 | - |
| PC aa C34:3* | Phosphatidylcholine with diacyl residue sum C34:3 | Phosphatidylcholines-diacyl | 0.504 | 0.298 | 0.855 | 0.011 | 0.03595 | - |
| PC aa C36:1* | Phosphatidylcholine with diacyl residue sum C36:1 | Phosphatidylcholines-diacyl | 0.507 | 0.296 | 0.869 | 0.0135 | 0.03595 | - |
| PC aa C36:2* | Phosphatidylcholine with diacyl residue sum C36:2 | Phosphatidylcholines-diacyl | 0.379 | 0.217 | 0.66 | 0.0006 | 0.00725 | - |
| PC aa C36:3* | Phosphatidylcholine with diacyl residue sum C36:3 | Phosphatidylcholines-diacyl | 0.468 | 0.281 | 0.778 | 0.0034 | 0.01446 | - |
| PC aa C38:4* | Phosphatidylcholine with diacyl residue sum C38:4 | Phosphatidylcholines-diacyl | 1.864 | 1.14 | 3.05 | 0.0131 | 0.03595 | + |
| PC aa C40:2* | Phosphatidylcholine with diacyl residue sum C40:2 | Phosphatidylcholines-diacyl | 0.441 | 0.267 | 0.728 | 0.0014 | 0.01164 | - |
| PC aa C40:3* | Phosphatidylcholine with diacyl residue sum C40:3 | Phosphatidylcholines-diacyl | 0.309 | 0.166 | 0.578 | 0.0002 | 0.00725 | - |
| PC aa C42:2* | Phosphatidylcholine with diacyl residue sum C42:2 | Phosphatidylcholines-diacyl | 0.456 | 0.271 | 0.769 | 0.0032 | 0.01446 | - |
| PC aa C42:4* | Phosphatidylcholine with diacyl residue sum C42:4 | Phosphatidylcholines-diacyl | 0.405 | 0.241 | 0.681 | 0.0007 | 0.00725 | - |
| SM (OH) C14:1* | Hydroxysphingomyelin with acyl residue sum C14:1 | Sphingomyelins | 0.486 | 0.29 | 0.814 | 0.0061 | 0.02127 | - |
| SM C24:0 | Sphingomyelin with acyl residue sum C24:0 | Sphingomyelins | 0.436 | 0.255 | 0.744 | 0.0023 | 0.01468 | - |
| SM C24:1 | Sphingomyelin with acyl residue sum C24:1 | Sphingomyelins | 0.424 | 0.239 | 0.75 | 0.0032 | 0.01468 | - |

Supp. Table S2b. Elevated Glucose in BLSA only

| Symbol | Full Name | Category | Odds Ratio Estimate | Lower 95% CI | Upper 95% CI | P value | FDR adjusted p value | Direction of Association |
| --- | --- | --- | --- | --- | --- | --- | --- | --- |
| Ala | Alanine | Amino acids | 2.485 | 1.398 | 4.418 | 0.0019 | 0.03744 | + |
| Ile* | Isoleucine | Amino acids | 2.093 | 1.243 | 3.524 | 0.0055 | 0.04148 | + |
| Pro* | Proline | Amino acids | 2.129 | 1.281 | 3.538 | 0.0036 | 0.03744 | + |
| DCA* | Deoxycholic Acid | Bile Acids | 1.609 | 1.093 | 2.367 | 0.0159 | 0.04996 | + |
| GCA* | Glycocholic Acid | Bile Acids | 1.736 | 1.128 | 2.671 | 0.0122 | 0.04996 | + |
| GDCA* | Glycodeoxycholic Acid | Bile Acids | 2.118 | 1.301 | 3.449 | 0.0025 | 0.02477 | + |
| PC ae C30:0* | Phosphatidylcholine with acyl-alkyl residue sum C30:0 | Phosphatidylcholines-acyl-alkyl | 0.482 | 0.283 | 0.819 | 0.007 | 0.01533 | - |
| PC ae C32:1* | Phosphatidylcholine with acyl-alkyl residue sum C32:1 | Phosphatidylcholines-acyl-alkyl | 0.363 | 0.197 | 0.67 | 0.0012 | 0.00503 | - |
| PC ae C32:2* | Phosphatidylcholine with acyl-alkyl residue sum C32:2 | Phosphatidylcholines-acyl-alkyl | 0.494 | 0.278 | 0.879 | 0.0164 | 0.02849 | - |
| PC ae C34:1* | Phosphatidylcholine with acyl-alkyl residue sum C34:1 | Phosphatidylcholines-acyl-alkyl | 0.363 | 0.198 | 0.667 | 0.0011 | 0.00503 | - |
| PC ae C34:2* | Phosphatidylcholine with acyl-alkyl residue sum C34:2 | Phosphatidylcholines-acyl-alkyl | 0.387 | 0.215 | 0.696 | 0.0015 | 0.0053 | - |
| PC ae C34:3* | Phosphatidylcholine with acyl-alkyl residue sum C34:3 | Phosphatidylcholines-acyl-alkyl | 0.322 | 0.171 | 0.605 | 0.0004 | 0.00503 | - |
| PC ae C36:1* | Phosphatidylcholine with acyl-alkyl residue sum C36:1 | Phosphatidylcholines-acyl-alkyl | 0.54 | 0.326 | 0.894 | 0.0167 | 0.02849 | - |
| PC ae C36:2* | Phosphatidylcholine with acyl-alkyl residue sum C36:2 | Phosphatidylcholines-acyl-alkyl | 0.341 | 0.182 | 0.638 | 0.0008 | 0.00503 | - |
| PC ae C36:3* | Phosphatidylcholine with acyl-alkyl residue sum C36:3 | Phosphatidylcholines-acyl-alkyl | 0.305 | 0.16 | 0.58 | 0.0003 | 0.00503 | - |
| PC ae C38:2* | Phosphatidylcholine with acyl-alkyl residue sum C38:2 | Phosphatidylcholines-acyl-alkyl | 0.403 | 0.228 | 0.714 | 0.0018 | 0.0053 | - |
| PC ae C38:3* | Phosphatidylcholine with acyl-alkyl residue sum C38:3 | Phosphatidylcholines-acyl-alkyl | 0.413 | 0.241 | 0.707 | 0.0013 | 0.00503 | - |
| PC ae C40:2* | Phosphatidylcholine with acyl-alkyl residue sum C40:2 | Phosphatidylcholines-acyl-alkyl | 0.516 | 0.318 | 0.836 | 0.0073 | 0.01533 | - |
| PC ae C40:3* | Phosphatidylcholine with acyl-alkyl residue sum C40:3 | Phosphatidylcholines-acyl-alkyl | 0.203 | 0.102 | 0.405 | <.0001 | 0.00023 | - |
| PC ae C40:5* | Phosphatidylcholine with acyl-alkyl residue sum C40:5 | Phosphatidylcholines-acyl-alkyl | 0.583 | 0.367 | 0.926 | 0.0222 | 0.03646 | - |
| PC ae C42:2* | Phosphatidylcholine with acyl-alkyl residue sum C42:2 | Phosphatidylcholines-acyl-alkyl | 0.396 | 0.222 | 0.707 | 0.0017 | 0.0053 | - |
| PC ae C42:3* | Phosphatidylcholine with acyl-alkyl residue sum C42:3 | Phosphatidylcholines-acyl-alkyl | 0.478 | 0.271 | 0.846 | 0.0112 | 0.02237 | - |
| PC ae C42:4* | Phosphatidylcholine with acyl-alkyl residue sum C42:4 | Phosphatidylcholines-acyl-alkyl | 0.469 | 0.286 | 0.769 | 0.0027 | 0.00736 | - |
| PC ae C42:5* | Phosphatidylcholine with acyl-alkyl residue sum C42:5 | Phosphatidylcholines-acyl-alkyl | 0.423 | 0.251 | 0.712 | 0.0012 | 0.00503 | - |
| PC ae C44:3* | Phosphatidylcholine with acyl-alkyl residue sum C44:3 | Phosphatidylcholines-acyl-alkyl | 0.498 | 0.307 | 0.808 | 0.0048 | 0.01171 | - |
| PC ae C44:4* | Phosphatidylcholine with acyl-alkyl residue sum C44:4 | Phosphatidylcholines-acyl-alkyl | 0.418 | 0.249 | 0.701 | 0.0009 | 0.00503 | - |
| PC ae C44:5* | Phosphatidylcholine with acyl-alkyl residue sum C44:5 | Phosphatidylcholines-acyl-alkyl | 0.398 | 0.223 | 0.708 | 0.0017 | 0.0053 | - |
| PC ae C44:6* | Phosphatidylcholine with acyl-alkyl residue sum C44:6 | Phosphatidylcholines-acyl-alkyl | 0.525 | 0.312 | 0.883 | 0.0152 | 0.02849 | - |
| SM (OH) C14:1* | Hydroxysphingomyelin with acyl residue sum C14:1 | Sphingomyelins | 0.432 | 0.251 | 0.743 | 0.0025 | 0.02539 | - |
| SM (OH) C16:1* | Hydroxysphingomyelin with acyl residue sum C16:1 | Sphingomyelins | 0.493 | 0.293 | 0.828 | 0.0075 | 0.02539 | - |
| SM (OH) C22:2* | Hydroxysphingomyelin with acyl residue sum C22:2 | Sphingomyelins | 0.417 | 0.224 | 0.775 | 0.0057 | 0.02539 | - |
| SM C16:0* | Sphingomyelin with acyl residue sum C16:0 | Sphingomyelins | 0.468 | 0.272 | 0.806 | 0.0062 | 0.02539 | - |

Supp. Table S2c. Reduced HDL in BLSA only

| Symbol | Full Name | Category | Odds Ratio Estimate | Lower 95% CI | Upper 95% CI | P value | FDR adjusted p value | Direction of Association |
| --- | --- | --- | --- | --- | --- | --- | --- | --- |
| N-C22:0-Cer* |  | Ceramides | 0.52 | 0.337 | 0.803 | 0.0032 | 0.01621 | - |
| N-C23:0-Cer* |  | Ceramides | 0.541 | 0.335 | 0.872 | 0.0117 | 0.02876 | - |
| N-C24:0-Cer* |  | Ceramides | 0.501 | 0.309 | 0.81 | 0.0049 | 0.01621 | - |
| N-C24:1-Cer* |  | Ceramides | 0.52 | 0.335 | 0.81 | 0.0038 | 0.01621 | - |
| N-C25:0-Cer* |  | Ceramides | 0.546 | 0.334 | 0.892 | 0.0157 | 0.03042 | - |
| PC ae C30:0* | Phosphatidylcholine with acyl-alkyl residue sum C30:0 | Phosphatidylcholines-acyl-alkyl | 0.461 | 0.279 | 0.762 | 0.0026 | 0.01314 | - |
| PC ae C32:2* | Phosphatidylcholine with acyl-alkyl residue sum C32:2 | Phosphatidylcholines-acyl-alkyl | 0.425 | 0.241 | 0.751 | 0.0032 | 0.01349 | - |
| PC ae C34:0* | Phosphatidylcholine with acyl-alkyl residue sum C34:0 | Phosphatidylcholines-acyl-alkyl | 0.607 | 0.395 | 0.933 | 0.0227 | 0.0427 | - |
| PC ae C34:1* | Phosphatidylcholine with acyl-alkyl residue sum C34:1 | Phosphatidylcholines-acyl-alkyl | 0.329 | 0.18 | 0.6 | 0.0003 | 0.00359 | - |
| PC ae C36:0* | Phosphatidylcholine with acyl-alkyl residue sum C36:0 | Phosphatidylcholines-acyl-alkyl | 0.542 | 0.348 | 0.847 | 0.0071 | 0.01677 | - |
| PC ae C36:2* | Phosphatidylcholine with acyl-alkyl residue sum C36:2 | Phosphatidylcholines-acyl-alkyl | 0.333 | 0.181 | 0.613 | 0.0004 | 0.00377 | - |
| PC ae C38:1* | Phosphatidylcholine with acyl-alkyl residue sum C38:1 | Phosphatidylcholines-acyl-alkyl | 0.482 | 0.285 | 0.815 | 0.0065 | 0.0166 | - |
| PC ae C40:3* | Phosphatidylcholine with acyl-alkyl residue sum C40:3 | Phosphatidylcholines-acyl-alkyl | 0.433 | 0.245 | 0.766 | 0.0041 | 0.01349 | - |
| PC ae C42:1* | Phosphatidylcholine with acyl-alkyl residue sum C42:1 | Phosphatidylcholines-acyl-alkyl | 0.531 | 0.324 | 0.871 | 0.0121 | 0.02359 | - |
| PC ae C42:2* | Phosphatidylcholine with acyl-alkyl residue sum C42:2 | Phosphatidylcholines-acyl-alkyl | 0.443 | 0.255 | 0.768 | 0.0037 | 0.01349 | - |
| PC ae C42:4* | Phosphatidylcholine with acyl-alkyl residue sum C42:4 | Phosphatidylcholines-acyl-alkyl | 0.542 | 0.344 | 0.854 | 0.0083 | 0.01895 | - |
| PC aa C30:0* | Phosphatidylcholine with diacyl residue sum C30:0 | Phosphatidylcholines-diacyl | 0.399 | 0.24 | 0.665 | 0.0004 | 0.00276 | - |
| PC aa C32:0* | Phosphatidylcholine with diacyl residue sum C32:0 | Phosphatidylcholines-diacyl | 0.353 | 0.19 | 0.654 | 0.0009 | 0.0039 | - |
| PC aa C32:2* | Phosphatidylcholine with diacyl residue sum C32:2 | Phosphatidylcholines-diacyl | 0.399 | 0.233 | 0.686 | 0.0009 | 0.0039 | - |
| PC aa C32:3* | Phosphatidylcholine with diacyl residue sum C32:3 | Phosphatidylcholines-diacyl | 0.388 | 0.202 | 0.746 | 0.0045 | 0.01325 | - |
| PC aa C34:1* | Phosphatidylcholine with diacyl residue sum C34:1 | Phosphatidylcholines-diacyl | 0.526 | 0.323 | 0.858 | 0.01 | 0.02753 | - |
| PC aa C34:2* | Phosphatidylcholine with diacyl residue sum C34:2 | Phosphatidylcholines-diacyl | 0.257 | 0.14 | 0.473 | <.0001 | 0.00027 | - |
| PC aa C34:3* | Phosphatidylcholine with diacyl residue sum C34:3 | Phosphatidylcholines-diacyl | 0.35 | 0.198 | 0.617 | 0.0003 | 0.00276 | - |
| PC aa C36:1* | Phosphatidylcholine with diacyl residue sum C36:1 | Phosphatidylcholines-diacyl | 0.395 | 0.224 | 0.698 | 0.0014 | 0.00517 | - |
| PC aa C36:2* | Phosphatidylcholine with diacyl residue sum C36:2 | Phosphatidylcholines-diacyl | 0.255 | 0.137 | 0.474 | <.0001 | 0.00027 | - |
| PC aa C36:3* | Phosphatidylcholine with diacyl residue sum C36:3 | Phosphatidylcholines-diacyl | 0.407 | 0.242 | 0.686 | 0.0007 | 0.0039 | - |
| PC aa C40:2* | Phosphatidylcholine with diacyl residue sum C40:2 | Phosphatidylcholines-diacyl | 0.475 | 0.296 | 0.763 | 0.0021 | 0.00714 | - |
| PC aa C40:3* | Phosphatidylcholine with diacyl residue sum C40:3 | Phosphatidylcholines-diacyl | 0.315 | 0.167 | 0.596 | 0.0004 | 0.00276 | - |
| PC aa C42:2* | Phosphatidylcholine with diacyl residue sum C42:2 | Phosphatidylcholines-diacyl | 0.552 | 0.341 | 0.895 | 0.016 | 0.04106 | - |
| SM C24:0* | Sphingomyelin with acyl residue sum C24:0 | Sphingomyelins | 0.481 | 0.285 | 0.812 | 0.0062 | 0.02921 | - |

Supp. Table S2d. Elevated Triglycerides in BLSA only

| Symbol | Full Name | Category | Odds Ratio Estimate | Lower 95% CI | Upper 95% CI | P value | FDR adjusted p value | Direction of Association |
| --- | --- | --- | --- | --- | --- | --- | --- | --- |
| alpha-AAA* | alpha-Aminoadipic acid | Biogenic amines | 1.995 | 1.308 | 3.044 | 0.0014 | 0.01319 | + |
| lysoPC a C18:0* | Lysophosphatidylcholine with acyl residue C18:0 | Lysophosphatidylcholines | 0.436 | 0.247 | 0.769 | 0.0042 | 0.02124 | - |
| lysoPC a C18:1* | Lysophosphatidylcholine with acyl residue C18:1 | Lysophosphatidylcholines | 0.537 | 0.331 | 0.87 | 0.0117 | 0.04008 | - |
| lysoPC a C18:2* | Lysophosphatidylcholine with acyl residue C18:2 | Lysophosphatidylcholines | 0.431 | 0.253 | 0.733 | 0.0019 | 0.02051 | - |
| PC ae C30:0* | Phosphatidylcholine with acyl-alkyl residue sum C30:0 | Phosphatidylcholines-acyl-alkyl | 0.431 | 0.257 | 0.725 | 0.0015 | 0.01314 | - |
| PC ae C30:1* | Phosphatidylcholine with acyl-alkyl residue sum C30:1 | Phosphatidylcholines-acyl-alkyl | 0.609 | 0.403 | 0.919 | 0.0182 | 0.03575 | - |
| PC ae C32:1* | Phosphatidylcholine with acyl-alkyl residue sum C32:1 | Phosphatidylcholines-acyl-alkyl | 0.325 | 0.173 | 0.611 | 0.0005 | 0.00593 | - |
| PC ae C32:2* | Phosphatidylcholine with acyl-alkyl residue sum C32:2 | Phosphatidylcholines-acyl-alkyl | 0.477 | 0.27 | 0.84 | 0.0104 | 0.02752 | - |
| PC ae C34:1* | Phosphatidylcholine with acyl-alkyl residue sum C34:1 | Phosphatidylcholines-acyl-alkyl | 0.391 | 0.216 | 0.706 | 0.0018 | 0.01314 | - |
| PC ae C36:2* | Phosphatidylcholine with acyl-alkyl residue sum C36:2 | Phosphatidylcholines-acyl-alkyl | 0.302 | 0.161 | 0.567 | 0.0002 | 0.00378 | - |
| PC ae C36:3 | Phosphatidylcholine with acyl-alkyl residue sum C36:3 | Phosphatidylcholines-acyl-alkyl | 0.402 | 0.223 | 0.727 | 0.0026 | 0.01314 | - |
| PC ae C38:1* | Phosphatidylcholine with acyl-alkyl residue sum C38:1 | Phosphatidylcholines-acyl-alkyl | 0.486 | 0.283 | 0.835 | 0.0089 | 0.02752 | - |
| PC ae C40:1* | Phosphatidylcholine with acyl-alkyl residue sum C40:1 | Phosphatidylcholines-acyl-alkyl | 0.52 | 0.31 | 0.872 | 0.0133 | 0.02752 | - |
| PC ae C40:3* | Phosphatidylcholine with acyl-alkyl residue sum C40:3 | Phosphatidylcholines-acyl-alkyl | 0.481 | 0.272 | 0.851 | 0.0119 | 0.02752 | - |
| PC ae C40:4 | Phosphatidylcholine with acyl-alkyl residue sum C40:4 | Phosphatidylcholines-acyl-alkyl | 0.563 | 0.343 | 0.923 | 0.0228 | 0.04221 | - |
| PC ae C42:1 | Phosphatidylcholine with acyl-alkyl residue sum C42:1 | Phosphatidylcholines-acyl-alkyl | 0.523 | 0.316 | 0.867 | 0.0119 | 0.02752 | - |
| PC ae C42:2* | Phosphatidylcholine with acyl-alkyl residue sum C42:2 | Phosphatidylcholines-acyl-alkyl | 0.484 | 0.279 | 0.837 | 0.0094 | 0.02752 | - |
| PC ae C42:3* | Phosphatidylcholine with acyl-alkyl residue sum C42:3 | Phosphatidylcholines-acyl-alkyl | 0.404 | 0.222 | 0.736 | 0.003 | 0.01314 | - |
| PC ae C42:4* | Phosphatidylcholine with acyl-alkyl residue sum C42:4 | Phosphatidylcholines-acyl-alkyl | 0.479 | 0.297 | 0.772 | 0.0025 | 0.01314 | - |
| PC ae C44:3 | Phosphatidylcholine with acyl-alkyl residue sum C44:3 | Phosphatidylcholines-acyl-alkyl | 0.58 | 0.368 | 0.912 | 0.0184 | 0.03575 | - |
| PC ae C44:4* | Phosphatidylcholine with acyl-alkyl residue sum C44:4 | Phosphatidylcholines-acyl-alkyl | 0.519 | 0.326 | 0.825 | 0.0055 | 0.01984 | - |
| PC aa C30:0* | Phosphatidylcholine with diacyl residue sum C30:0 | Phosphatidylcholines-diacyl | 0.506 | 0.312 | 0.823 | 0.0061 | 0.03341 | - |
| PC aa C32:2* | Phosphatidylcholine with diacyl residue sum C32:2 | Phosphatidylcholines-diacyl | 0.458 | 0.27 | 0.776 | 0.0037 | 0.02978 | - |
| PC aa C34:2* | Phosphatidylcholine with diacyl residue sum C34:2 | Phosphatidylcholines-diacyl | 0.378 | 0.215 | 0.664 | 0.0007 | 0.01242 | - |
| PC aa C34:3* | Phosphatidylcholine with diacyl residue sum C34:3 | Phosphatidylcholines-diacyl | 0.486 | 0.285 | 0.831 | 0.0084 | 0.03567 | - |
| PC aa C36:2* | Phosphatidylcholine with diacyl residue sum C36:2 | Phosphatidylcholines-diacyl | 0.329 | 0.182 | 0.594 | 0.0002 | 0.00812 | - |
| PC aa C36:3 | Phosphatidylcholine with diacyl residue sum C36:3 | Phosphatidylcholines-diacyl | 0.54 | 0.329 | 0.886 | 0.0146 | 0.04534 | - |
| PC aa C40:2* | Phosphatidylcholine with diacyl residue sum C40:2 | Phosphatidylcholines-diacyl | 0.458 | 0.281 | 0.745 | 0.0016 | 0.0191 | - |
| PC aa C40:3* | Phosphatidylcholine with diacyl residue sum C40:3 | Phosphatidylcholines-diacyl | 0.416 | 0.227 | 0.762 | 0.0045 | 0.02978 | - |
| PC aa C42:2* | Phosphatidylcholine with diacyl residue sum C42:2 | Phosphatidylcholines-diacyl | 0.507 | 0.305 | 0.84 | 0.0084 | 0.03567 | - |
| PC aa C42:4 | Phosphatidylcholine with diacyl residue sum C42:4 | Phosphatidylcholines-diacyl | 0.535 | 0.327 | 0.875 | 0.0126 | 0.04269 | - |

Supp. Table S2e. Elevated Waist Circumference in BLSA only

| Symbol | Full Name | Category | Odds Ratio Estimate | Lower 95% CI | Upper 95% CI | P value | FDR adjusted p value | Direction of Association |
| --- | --- | --- | --- | --- | --- | --- | --- | --- |
| C5* | Isovalerylcarnitine / 2-Methylbutyrylcarnitine / Valerylcarnitine | acylcarnitines | 2.192 | 1.327 | 3.623 | 0.0022 | 0.01855 | + |
| C16* | Hexadecanoylcarnitine [= Palmitoylcarnitine] | acylcarnitines | 2.129 | 1.291 | 3.512 | 0.0031 | 0.01855 | + |
| Ile* | Isoleucine | Amino acids | 2.322 | 1.373 | 3.924 | 0.0017 | 0.03506 | + |
| N-C18:0-Cer* |  | Ceramides | 2.98 | 1.718 | 5.169 | 0.0001 | 0.00104 | + |
| N-C20:0-Cer* |  | Ceramides | 2.031 | 1.227 | 3.362 | 0.0059 | 0.0298 | + |
| H1* | Glucose | hexoses | 1.83 | 1.141 | 2.936 | 0.0122 | 0.01234 | + |
| lysoPC a C18:1* | Lysophosphatidylcholine with acyl residue C18:1 | Lysophosphatidylcholines | 0.442 | 0.259 | 0.753 | 0.0027 | 0.01894 | - |
| lysoPC a C18:2* | Lysophosphatidylcholine with acyl residue C18:2 | Lysophosphatidylcholines | 0.442 | 0.256 | 0.764 | 0.0035 | 0.01894 | - |
| PC ae C30:1 | Phosphatidylcholine with acyl-alkyl residue sum C30:1 | Phosphatidylcholines-acyl-alkyl | 0.59 | 0.372 | 0.935 | 0.0249 | 0.04684 | - |
| PC ae C34:1* | Phosphatidylcholine with acyl-alkyl residue sum C34:1 | Phosphatidylcholines-acyl-alkyl | 0.521 | 0.292 | 0.931 | 0.0276 | 0.04991 | - |
| PC ae C34:2* | Phosphatidylcholine with acyl-alkyl residue sum C34:2 | Phosphatidylcholines-acyl-alkyl | 0.396 | 0.218 | 0.719 | 0.0024 | 0.02154 | - |
| PC ae C34:3* | Phosphatidylcholine with acyl-alkyl residue sum C34:3 | Phosphatidylcholines-acyl-alkyl | 0.46 | 0.257 | 0.825 | 0.0091 | 0.03502 | - |
| PC ae C36:2* | Phosphatidylcholine with acyl-alkyl residue sum C36:2 | Phosphatidylcholines-acyl-alkyl | 0.336 | 0.178 | 0.636 | 0.0008 | 0.00976 | - |
| PC ae C36:3* | Phosphatidylcholine with acyl-alkyl residue sum C36:3 | Phosphatidylcholines-acyl-alkyl | 0.434 | 0.236 | 0.799 | 0.0073 | 0.03326 | - |
| PC ae C38:1* | Phosphatidylcholine with acyl-alkyl residue sum C38:1 | Phosphatidylcholines-acyl-alkyl | 0.522 | 0.31 | 0.878 | 0.0142 | 0.03747 | - |
| PC ae C38:2* | Phosphatidylcholine with acyl-alkyl residue sum C38:2 | Phosphatidylcholines-acyl-alkyl | 0.336 | 0.184 | 0.612 | 0.0004 | 0.00823 | - |
| PC ae C40:1 | Phosphatidylcholine with acyl-alkyl residue sum C40:1 | Phosphatidylcholines-acyl-alkyl | 0.521 | 0.311 | 0.872 | 0.0131 | 0.03747 | - |
| PC ae C40:3* | Phosphatidylcholine with acyl-alkyl residue sum C40:3 | Phosphatidylcholines-acyl-alkyl | 0.483 | 0.27 | 0.864 | 0.0141 | 0.03747 | - |
| PC ae C40:5* | Phosphatidylcholine with acyl-alkyl residue sum C40:5 | Phosphatidylcholines-acyl-alkyl | 0.567 | 0.355 | 0.905 | 0.0173 | 0.0395 | - |
| PC ae C40:6* | Phosphatidylcholine with acyl-alkyl residue sum C40:6 | Phosphatidylcholines-acyl-alkyl | 0.466 | 0.277 | 0.783 | 0.004 | 0.02372 | - |
| PC ae C42:2* | Phosphatidylcholine with acyl-alkyl residue sum C42:2 | Phosphatidylcholines-acyl-alkyl | 0.48 | 0.275 | 0.837 | 0.0098 | 0.03502 | - |
| PC ae C42:3* | Phosphatidylcholine with acyl-alkyl residue sum C42:3 | Phosphatidylcholines-acyl-alkyl | 0.325 | 0.173 | 0.609 | 0.0005 | 0.00823 | - |
| PC ae C42:4* | Phosphatidylcholine with acyl-alkyl residue sum C42:4 | Phosphatidylcholines-acyl-alkyl | 0.481 | 0.289 | 0.801 | 0.0049 | 0.02526 | - |
| PC ae C42:5 | Phosphatidylcholine with acyl-alkyl residue sum C42:5 | Phosphatidylcholines-acyl-alkyl | 0.542 | 0.331 | 0.886 | 0.0146 | 0.03747 | - |
| PC ae C44:3* | Phosphatidylcholine with acyl-alkyl residue sum C44:3 | Phosphatidylcholines-acyl-alkyl | 0.474 | 0.286 | 0.786 | 0.0038 | 0.02372 | - |
| PC ae C44:4 | Phosphatidylcholine with acyl-alkyl residue sum C44:4 | Phosphatidylcholines-acyl-alkyl | 0.554 | 0.339 | 0.906 | 0.0186 | 0.0395 | - |
| PC ae C44:5 | Phosphatidylcholine with acyl-alkyl residue sum C44:5 | Phosphatidylcholines-acyl-alkyl | 0.514 | 0.296 | 0.893 | 0.0181 | 0.0395 | - |
| PC ae C44:6 | Phosphatidylcholine with acyl-alkyl residue sum C44:6 | Phosphatidylcholines-acyl-alkyl | 0.546 | 0.324 | 0.922 | 0.0235 | 0.04684 | - |
| PC aa C38:0 | Phosphatidylcholine with diacyl residue sum C38:0 | Phosphatidylcholines-diacyl | 0.436 | 0.244 | 0.779 | 0.0051 | 0.04138 | - |
| PC aa C38:1 | Phosphatidylcholine with diacyl residue sum C38:1 | Phosphatidylcholines-diacyl | 0.429 | 0.233 | 0.789 | 0.0064 | 0.04138 | - |
| PC aa C40:2 | Phosphatidylcholine with diacyl residue sum C40:2 | Phosphatidylcholines-diacyl | 0.508 | 0.309 | 0.833 | 0.0073 | 0.04138 | - |
| PC aa C40:3 | Phosphatidylcholine with diacyl residue sum C40:3 | Phosphatidylcholines-diacyl | 0.439 | 0.245 | 0.789 | 0.0059 | 0.04138 | - |
| PC aa C42:0 | Phosphatidylcholine with diacyl residue sum C42:0 | Phosphatidylcholines-diacyl | 0.479 | 0.284 | 0.807 | 0.0057 | 0.04138 | - |
| PC aa C42:1 | Phosphatidylcholine with diacyl residue sum C42:1 | Phosphatidylcholines-diacyl | 0.489 | 0.292 | 0.817 | 0.0063 | 0.04138 | - |

Supp. Table S2f. Elevated Blood Pressure in BLSA only

| Symbol | Full Name | Category | Odds Ratio Estimate | Lower 95% CI | Upper 95% CI | P value | FDR adjusted p value | Direction of Association |
| --- | --- | --- | --- | --- | --- | --- | --- | --- |
| c-C18:2w6 | Linoleic acid | Free Fatty Acid | 0.469 | 0.276 | 0.799 | 0.0054 | 0.04291 | - |
| H1* | Glucose | hexoses | 1.845 | 1.149 | 2.962 | 0.0112 | 0.01104 | + |
| PC ae C32:1* | Phosphatidylcholine with acyl-alkyl residue sum C32:1 | Phosphatidylcholines-acyl-alkyl | 0.366 | 0.2 | 0.669 | 0.0011 | 0.03536 | - |
| PC ae C34:3 | Phosphatidylcholine with acyl-alkyl residue sum C34:3 | Phosphatidylcholines-acyl-alkyl | 0.457 | 0.259 | 0.805 | 0.0067 | 0.04967 | - |
| PC ae C36:2* | Phosphatidylcholine with acyl-alkyl residue sum C36:2 | Phosphatidylcholines-acyl-alkyl | 0.44 | 0.242 | 0.799 | 0.007 | 0.04967 | - |
| PC ae C38:2* | Phosphatidylcholine with acyl-alkyl residue sum C38:2 | Phosphatidylcholines-acyl-alkyl | 0.432 | 0.248 | 0.752 | 0.003 | 0.03536 | - |
| PC ae C44:3* | Phosphatidylcholine with acyl-alkyl residue sum C44:3 | Phosphatidylcholines-acyl-alkyl | 0.473 | 0.29 | 0.773 | 0.0028 | 0.03536 | - |
| PC aa C32:0* | Phosphatidylcholine with diacyl residue sum C32:0 | Phosphatidylcholines-diacyl | 0.441 | 0.241 | 0.807 | 0.008 | 0.04553 | - |
| PC aa C34:2* | Phosphatidylcholine with diacyl residue sum C34:2 | Phosphatidylcholines-diacyl | 0.399 | 0.229 | 0.694 | 0.0011 | 0.01932 | - |
| PC aa C36:2* | Phosphatidylcholine with diacyl residue sum C36:2 | Phosphatidylcholines-diacyl | 0.417 | 0.239 | 0.727 | 0.002 | 0.01973 | - |
| PC aa C40:2* | Phosphatidylcholine with diacyl residue sum C40:2 | Phosphatidylcholines-diacyl | 0.403 | 0.237 | 0.686 | 0.0008 | 0.01932 | - |
| PC aa C42:2* | Phosphatidylcholine with diacyl residue sum C42:2 | Phosphatidylcholines-diacyl | 0.43 | 0.25 | 0.74 | 0.0023 | 0.01973 | - |
| PC aa C42:4* | Phosphatidylcholine with diacyl residue sum C42:4 | Phosphatidylcholines-diacyl | 0.493 | 0.301 | 0.806 | 0.0048 | 0.03228 | - |

Supp. Table 2g. MetS in TMCS only

| Symbol | Full Name | Category | Odds Ratio Estimate | Lower 95% CI | Upper 95% CI | P value | FDR adjusted p value | Direction of Association |
| --- | --- | --- | --- | --- | --- | --- | --- | --- |
| Val* | Valine | Amino acids | 2.584 | 1.419 | 4.707 | 0.0019 | 0.04013 | + |
| SDMA* | Symmetric dimethylarginine | Biogenic amines | 0.367 | 0.183 | 0.735 | 0.0047 | 0.04683 | - |
| c-C20:3w6* | cis-8,11,14-Eicosatrienoic acid | Free Fatty Acid | 2.992 | 1.445 | 6.193 | 0.0032 | 0.02525 | + |

Supp. Table S2h. Elevated Triglycerides in TMCS only

| Symbol | Full Name | Category | Odds Ratio Estimate | Lower 95% CI | Upper 95% CI | P value | FDR adjusted p value | Direction of Association |
| --- | --- | --- | --- | --- | --- | --- | --- | --- |
| SM (OH) C14:1* | Hydroxysphingomyelin with acyl residue sum C14:1 | Sphingomyelins | 0.471 | 0.255 | 0.87 | 0.0161 | 0.04086 | - |
| SM (OH) C16:1* | Hydroxysphingomyelin with acyl residue sum C16:1 | Sphingomyelins | 0.501 | 0.269 | 0.933 | 0.0293 | 0.04564 | - |
| SM (OH) C22:2* | Hydroxysphingomyelin with acyl residue sum C22:2 | Sphingomyelins | 0.361 | 0.152 | 0.854 | 0.0204 | 0.04086 | - |
| SM (OH) C24:1* | Hydroxysphingomyelin with acyl residue sum C24:1 | Sphingomyelins | 0.35 | 0.172 | 0.71 | 0.0037 | 0.0171 | - |
| SM C24:0* | Sphingomyelin with acyl residue sum C24:0 | Sphingomyelins | 0.478 | 0.264 | 0.865 | 0.0148 | 0.04086 | - |
| SM C24:1* | Sphingomyelin with acyl residue sum C24:1 | Sphingomyelins | 0.316 | 0.163 | 0.613 | 0.0007 | 0.00722 | - |
| SM C26:0* | Sphingomyelin with acyl residue sum C26:0 | Sphingomyelins | 0.44 | 0.22 | 0.88 | 0.0203 | 0.04086 | - |
| SM C26:1* | Sphingomyelin with acyl residue sum C26:1 | Sphingomyelins | 0.513 | 0.284 | 0.924 | 0.0263 | 0.04564 | - |

TMCS had no unique significant associations for elevated glucose, waist circumference, and blood pressure and for reduced HDL.

*Associations that remain significant after adjustment with additional covariates smoking and DASH score.

Negative (-) Direction of Association: Odds Ratio < 1 at 0.05 significance level; Positive (+) Direction of Association: Odds Ratio > 1 at 0.05 significance level.

Multivariate logistic regression models were used to test for significance and direction.

BLSA: Baltimore Longitudinal Study of Aging; TMCS: Tsuruoka Metabolomics Cohort Study; FDR: False Discovery Rate.

Supplementary Text

Methods

*Blood Samples*

BLSA: Venous blood samples were collected between 6 and 7 AM following an overnight fast. Serum samples were aliquoted into 0.5-mL volume in Nunc cryogenic tubes and stored at −80 ̊C until further use. Samples were not subject to any freeze–thaw cycles prior to metabolomic assays. The average storage time of serum samples for quantitative metabolomics in BLSA participants was 4.36 years (SD: 2.33) (Table 1).

TMCS: Venous blood samples were collected between 8:30 and 10:30 AM following an overnight fast. Serum samples were collected with serum-separating medium and stored at room temperature after collection. Serum samples for quantitative metabolomics were assayed during ongoing recruitment; storage time was <6 hours and did not vary across TMCS participants.

*Other outcomes*

MetS was defined using the Third Adults Treatment Panel of the National Cholesterol Education Program (NCEPATPIII) criteria, revised by the American Heart Association and National Heart, Lung, and Blood Institute (AHA/NHLBI) [1]

BLSA: plasma triglycerides and fasting plasma glucose were analyzed using enzymatic methods detailed previously [2, 3]. HDL-C values were measured by a dextran sulfate–magnesium precipitation procedure. Waist circumference was measured by BLSA staff clinicians with a tape measure kept parallel to the floor, from the hip bone and wrapping around the waist at the level of the umbilicus while participants were holding their breath [4]. Systolic and diastolic blood pressures were each measured three times in both arms in a seated position using a mercury sphygmomanometer appropriately sized to the arm of the participant, and the mean of the systolic and diastolic measurements were used in analysis [5].

TMCS: Serum levels of triglycerides, and fasting plasma glucose were analyzed using Hexokinase methods [6]. HDL-C values were measured by a direct method. Waist circumference was measured to the nearest 0.1 cm at the umbilicus at the end of a normal breath. If the umbilicus drooped down, the measurement was made midway between the inferior margin of the last rib and the top of the iliac crest in a horizontal plane [7]. Systolic and diastolic blood pressures were each measured twice on one occasion in a seated position using an automated sphygmomanometer (Omron HBP-T105S-N), and the mean of each of the two measurements were used in analysis [7].

*Quantitative metabolomics - BLSA*

AbsoluteIDQ p180: This commercially available platform allows for the quantification of amino acids, acylcarnitines, sphingomyelins, phosphatidylcholines (including both diacyl and acyl-alkyl residues), hexoses, and biogenic amines. Briefly, the validated assay is based on phenylisothiocyanate (PITC) derivatization in the presence of stable isotope-labeled internal standards, followed by two different mass spectrometric methods for absolute quantification of metabolites. The acylcarnitines, lipids, and hexoses were analyzed by flow injection analysis-tandem mass spectrometry (FIA-MS/MS). The amino acids and biogenic amines were analyzed by liquid chromatography tandem-mass spectrometry (LC-MS/MS) using an AB SCIEX 4000 QTrap^®^ mass spectrometer (AB SCIEX, Darmstadt, Germany) with electrospray ionization (ESI). Calculation of analyte concentrations, data assessment and compilation was performed using MetIDQ software (Biocrates Life Sciences AG).

Bile acids: The quantification of bile acids in the BLSA samples was performed with the Biocrates Bile Acids kit. A highly selective reversed phase LC-MS/MS analysis method in negative ion multiple reaction monitoring (MRM) detection mode was applied to determine the concentrations of bile acids. The samples were extracted via dried filter spot technique in 96-well plate format. Sample extracts were measured by LC-ESI-MS/MS with a tandem mass spectrometry instrument (Thermo Fisher Scientific TSQ). For highly accurate quantification, 7-point external calibration curves and 10 stable isotope-labeled internal standards were applied. Data of bile acids were quantified using the appropriate MS software (Thermo Fisher Scientific Xcalibur^TM^) and the results were imported into Biocrates MetIDQ software.

Lipids: Targeted lipid metabolomics in BLSA samples were performed after derivatization to their corresponding methyl ester derivatives (FAMES). Free fatty acids were determined by gas chromatography-tandem mass spectrometry (GC-MS/MS) using Agilent 7890 GC/5975 MSD (Agilent, Santa Clara, USA) instruments. Samples were treated with methanolic hydrochloric acid solution for 45 minutes at 25°C to completely convert fatty acids into their methyl esters. After neutralization with sodium carbonate, samples were extracted with hexane. Chromatograms were recorded in selected ion monitoring (SIM) mode. External standard calibration curves and five internal standards were used to calculate concentrations of individual free fatty acids. Data were quantified using the appropriate mass spectrometry software (Agilent MassHunter^TM^) and imported into Biocrates MetIDQ^TM^ software to calculate analyte concentrations, data assessment and compilation. In a separate approach, the biologically most abundant phospho- and sphingolipids not quantified in the p180 kit, including (lyso‑) phosphatidylglycerols, (lyso-) phosphatidylethanolamines, phosphatidylserines, ceramides, dihydroceramides, and 2-hydroxyacylceramides, were quantitatively analyzed by high-throughput FIA-ESI-MS/MS. The reported lipid annotation represents a sum signal of all isobaric lipids with the same molecular weight (±0.5 Da range) within the same lipid class. MRM detection in positive and negative mode was performed using a SCIEX 4000 QTRAP^®^ (AB SCIEX, Darmstadt, Germany) instrument. Samples were extracted by MeOH/CHCl3-liquid/liquid-extraction. In addition to five internal standards to compensate for matrix effects, 43 external standards were used for a multi-point calibration. Calculation of analyte concentrations, data assessment and compilation was performed with MetIDQ™ software enabling isotopic correction.

*Quantitative metabolomics – TMCS*

MxP^®^ Quant 500: Lipids were measured by FIA-MS/MS using a 5500 QTRAP^®^ instrument (AB Sciex, Darmstadt, Germany) with an ESI source, and small molecules including bile acids were measured by LC-MS/MS using the same 5500 QTRAP^®^ instrument. The experimental metabolomics measurement technique is described in detail by patents EP1897014B1 and EP1875401B1 (accessible online at https://patents.google.com/patent/EP1897014B1 and https://patents.google.com/patent/EP1875401B1). Briefly, a 96-well based sample preparation device was used to quantitatively analyze the metabolite profile in the samples. This device consists of inserts that have been impregnated with internal standards, and a predefined sample amount was added to the inserts. Next, a PITC solution was added to derivatize some of the analytes (e.g. amino acids), and after the derivatization was completed, the target analytes were extracted with an organic solvent, followed by a dilution step. The obtained extracts were then analyzed by FIA-MS/MS and LC-MS/MS methods using MRM to detect the analytes. Data were quantified using appropriate mass spectrometry software (Sciex Analyst^®^) and imported into Biocrates MetIDQ™ software for calculating analyte concentrations, data assessment and compilation.

1. Gomez, G., et al., *Metabolic Syndrome and Amyloid Accumulation in the Aging Brain.* J Alzheimers Dis, 2018. **65**(2): p. 629-639.

2. Metter, E.J., et al., *Glucose and insulin measurements from the oral glucose tolerance test and mortality prediction.* Diabetes Care, 2008. **31**(5): p. 1026-30.

3. Mielke, M.M., et al., *Factors affecting longitudinal trajectories of plasma sphingomyelins: the Baltimore Longitudinal Study of Aging.* Aging Cell, 2015. **14**(1): p. 112-21.

4. Beydoun, M.A., et al., *Vitamin D receptor and megalin gene polymorphisms are associated with central adiposity status and changes among US adults.* J Nutr Sci, 2013. **2**: p. e33.

5. Brant, L.J., et al., *Gender differences in the accuracy of time-dependent blood pressure indices for predicting coronary heart disease: A random-effects modeling approach.* Gend Med, 2010. **7**(6): p. 616-27.

6. Nakamura, M., et al., *Comparison between the triglycerides standardization of routine methods used in Japan and the chromotropic acid reference measurement procedure used by the CDC Lipid Standardization Programme.* Ann Clin Biochem, 2016. **53**(6): p. 632-639.

7. Iida, M., et al., *Profiling of plasma metabolites in postmenopausal women with metabolic syndrome.* Menopause, 2016. **23**(7): p. 749-58.
